# CAG repeat expansion in the Huntington’s disease gene shapes linear and circular RNAs biogenesis

**DOI:** 10.1371/journal.pgen.1010988

**Published:** 2023-10-13

**Authors:** Dilara Ayyildiz, Guendalina Bergonzoni, Alan Monziani, Takshashila Tripathi, Jessica Döring, Emanuela Kerschbamer, Francesca Di Leva, Elia Pennati, Luisa Donini, Marina Kovalenko, Jacopo Zasso, Luciano Conti, Vanessa C. Wheeler, Christoph Dieterich, Silvano Piazza, Erik Dassi, Marta Biagioli

**Affiliations:** 1 Bioinformatic facility, Department of Cellular, Computational and Integrative Biology, CIBIO, University of Trento, Trento, Italy; 2 Biomedical Sciences and Biotechnology, University of Udine, Udine, Italy; 3 NeuroEpigenetics laboratory, Department of Cellular, Computational and Integrative Biology, CIBIO, University of Trento, Trento, Italy; 4 Molecular Neurogenetics Unit, Center for Genomic Medicine, Massachusetts General Hospital, Boston, Massachusetts, United States of America; 5 Laboratory of Stem Cell Biology, Department of Cellular, Computational and Integrative Biology, CIBIO, University of Trento, Trento, Italy; 6 Department of Neurology Harvard Medical School, Boston, Massachusetts, United States of America; 7 Section of Bioinformatics and Systems Cardiology, University Hospital Heidelberg, Heidelberg, Germany; 8 Laboratory of RNA Regulatory Networks, Department of Cellular, Computational and Integrative Biology, CIBIO, University of Trento, Trento, Italy; Case Western Reserve University, UNITED STATES

## Abstract

Alternative splicing (AS) appears to be altered in Huntington’s disease (HD), but its significance for early, pre-symptomatic disease stages has not been inspected. Here, taking advantage of *Htt* CAG knock-in mouse *in vitro* and *in vivo* models, we demonstrate a correlation between *Htt* CAG repeat length and increased aberrant linear AS, specifically affecting neural progenitors and, *in vivo*, the striatum prior to overt behavioral phenotypes stages. Remarkably, a significant proportion (36%) of the aberrantly spliced isoforms are not-functional and meant to non-sense mediated decay (NMD). The expanded *Htt* CAG repeats further reflect on a previously neglected, global impairment of back-splicing, leading to decreased circular RNAs production in neural progenitors. Integrative transcriptomic analyses unveil a network of transcriptionally altered micro-RNAs and RNA-binding proteins (Celf, hnRNPs, Ptbp, Srsf, Upf1, Ythd2) which might influence the AS machinery, primarily in neural cells. We suggest that this unbalanced expression of linear and circular RNAs might alter neural fitness, contributing to HD pathogenesis.

## Author summary

This study establishes the first evidence for a direct correlation between *Htt* CAG repeat expansion and altered RNA splicing in neurons. Specifically, we demonstrate that accurate murine model systems, mimicking the human HD mutation, exhibit a mis-shaped repertoire of linear and back-spliced circular RNA isoforms. Mechanistically, our integrative analysis points to aberrantly regulated miRNAs and RNA-binding proteins implicated in NMD, M6A modification and splicing regulation, thus unveiling a possible new angle of HD biology. To conclude, this work contributes to the understanding of neuronal and striatal vulnerability in HD pathogenesis and delineates new potential molecular targets for therapeutic intervention.

## Introduction

Huntington’s disease (HD) is a hereditary, fatal neurodegenerative disorder caused by a CAG trinucleotide expansion within exon 1 of the *HTT* gene [1]. Explicit clinical onset typically occurs in mid-life and leads to an inexorable decline to death after 10–15 years [2]. A polymorphic CAG tract up to 35 repeats is found in unaffected individuals, whereas alleles bearing 36 or more repeats lead to HD symptoms. Since the *HTT* gene is ubiquitously expressed during human development and in all body districts, the effects of the mutation are strongly pleiotropic [3]. The central nervous system, however, remains the main region affected by mutant *HTT*, with a prominent loss of GABAergic medium-sized spiny neurons of the striatum, constituting the major contributor to movement, cognitive and behavioral dysfunctions [4–6]. So far, the rate-limiting mechanism(s) of neurodegeneration remains elusive although chromatin, transcription, and RNA processing dysregulations are emerging as fundamental features [3,7]. In particular, RNA processing and alternative splicing (AS) alterations might affect the level and composition of a broad repertoire of proteins, thus contributing to HD striatal vulnerability and pathogenesis. The spliceosomal activity can be directly modulated by the expression level and/or sequestration of various proteins that bind to nascent mRNA. Interestingly, huntingtin can associate with the WW-containing proteins HYPA and HYPC (*Htt* Yeast two-hybrid Protein A and C), also known as FBP11/PRPF40A, and PRPF40B, respectively [8–10], participating in early spliceosomal assembly and 5’ site recognition. On the other hand, mis-splicing events in individuals with highly expanded *HTT* CAG repeats have been shown to produce the small, highly pathogenic, polyadenylated exon 1-intron 1 *HTT* transcript [11]. Moreover, tissue-restricted trans-splicing regulators, binding auxiliary exonic and intronic cis-regulatory signals, modulate splice-site choice by interacting with components of the splicing machinery [12,13]. Coherently, recent evidence supports a role for mutant huntingtin in dysregulating the expression of four RNA-binding proteins (PTBP1, SFRS4, RBM4, SREK1) in HD *post mortem* brains, thus, correlating with abnormal splicing in the central nervous system [14].

As experimental evidence points toward dysregulated AS as a key feature in HD pathology, it is tempting to hypothesize that nuclear mutant huntingtin might alter AS outcome through different direct and/or indirect modes, affecting the relative abundance of target proteins, but also producing dysfunctional isoforms which could be targeted to nonsense-mediated decay (NMD) [15].

Importantly, AS regulation is crucial not only to the establishment of a repertoire of protein-coding isoforms extremely relevant for the proper physiology of the nervous system, but also to the biogenesis of circular RNAs (circRNAs), unusually stable non-coding RNAs produced by the circularization of exons [16–18]. CircRNAs are highly enriched in neurons and have been implicated in a wide variety of pathological conditions, including neurological diseases [19]. CircRNAs biogenesis seems to correlate with exon-skipping events [20]. However, several RNA-binding proteins (such as QKI, hnRNPL, FUS) have been described to regulate exons circularization, while RNA editing processes–conversion of adenosine-to-inosine (ADAR1) and N6-methyladenosine (m6A) RNA modification, were recently associated with inhibition or promotion of circRNA biogenesis, respectively [21,22]. Notwithstanding, the exact mechanisms of spliced exon-exon circularization remains unclear. The vast majority of circular RNAs localizes in the cytoplasm and their exonic sequences might encompass RNAs (miRNAs) and RNA-Binding Proteins (RBPs) binding sites [17,23], thus acting as competing-endogenous RNA [23]. In this scenario, a circRNA could efficiently engage (“sponge”) either miRNAs or RBPs, or both, eventually relieving their canonical mRNA targets from post-transcriptional regulation [17,24–27]. Whether mutant huntingtin may modulate circular RNA expression levels remains unexplored.

Here, we investigated whether linear and back-splicing processes, producing linear mRNAs and circular isoforms, might be altered by the HD mutation in a CAG-dependent and neural-specific manner. We unveiled new mechanisms dysregulated in HD at the genome-wide level and possible regulatory cross talks with miRNAs, NMD, RNA modifications and RBPs.

## Results

### Aberrant linear alternative splicing shows *Htt* CAG length and age dependency, specifically in the mouse striatum

In order to gauge evidence of AS alterations in the brain regions of HD animal models, we examined publicly available RNA sequencing (RNAseq) datasets (striatum (GSE65774), cortex (GSE65770) and liver (GSE65772), available through HDinHD, (see also Methods) [28]. The AS events in striatum, cortex and liver of 6 knock-in (KI) mouse models (Q20, Q80, Q92, Q111, Q140 and Q175) carrying different *Htt* CAG repeat lengths were determined using rMATS [29,30]. Q20 mice, with the lowest *Htt* CAG repeat length, were used as controls. The total number of genes showing one of the five major types of AS events [alternative 5’ splice site (A5SS), alternative 3’ splice site (A3SS), mutually exclusive exons (MXE), retained intron (RI) and skipped exon (SE)] was evaluated using a collection of both reads spanning splicing junctions (JC) and reads spanning splicing junctions plus on target (JCEC) (S1 Table).

**Fig 1:**
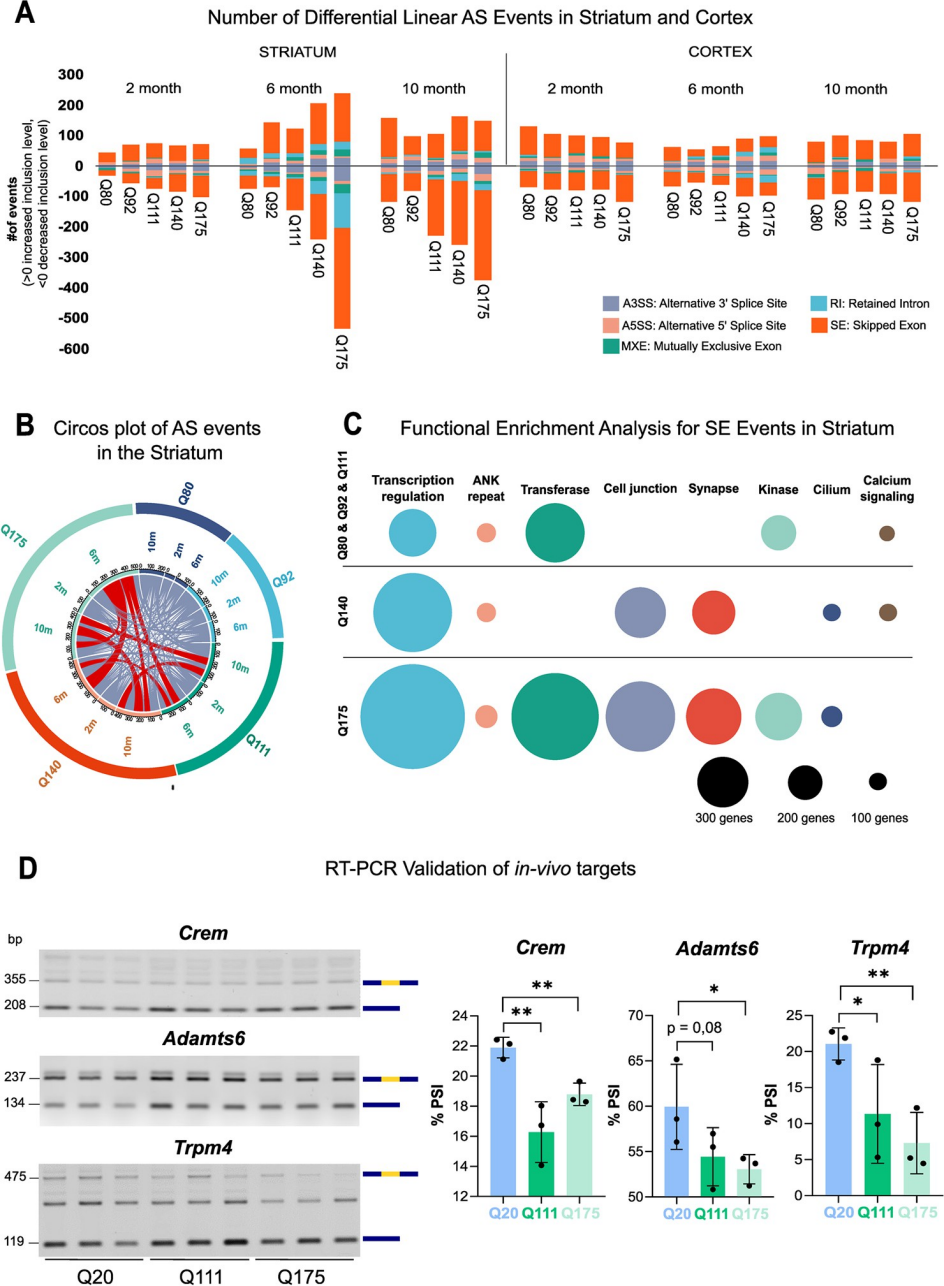
Aberrant linear alternative splicing in the striatum of KI animal models of HD. **A)** Bar graphs show the number of differential AS events in the striatum and cortex from 6 mouse KI models (Q20, Q80, Q92, Q111, Q140 and Q175) of HD, presenting different *Htt* CAG repeat lengths and 3 ages (2, 6, and 10 months). The number of events is shown for each genotype, time point and brain region. The inclusion level is calculated in comparison to Q20 controls and the positive or negative values are plotted. Source data by Langfelder P. *et al* (2016) [28]. Further details can be found in the Methods section. Each color of the bar chart represents a different AS type. **B)** Circos plot represents the number of transcripts within the striatum—showing differential AS events—shared between different genotypes (Q80, Q92, Q111, Q140 and Q175) and time points (2, 6, and 10 months). Conditions (genotypes and/or time points) sharing more than 50 transcripts are depicted in red. **C)** Weighted nodes graphical representation shows the functional enrichment analysis for transcripts displaying significant skipped exon (SE) events in the striatum. Highly expanded *Htt* CAG sizes (Q140 and Q175), the major contributors to aberrant SE in the striatum, are shown separated. Nodes’ size legend is depicted at the bottom. **D)** Representative agarose gel images (left) and quantification plots (right) report the RT-PCR results of AS validation for *Crem*, *Adamts6*, and *Trpm4*, selected transcript targets. RT-PCR assay and quantification were performed on striata RNA from an independent set of wild-type (WT), Q20 and Q111/Q175 mice. Transcripts isoforms with inclusion or exclusion of the variable exon (in yellow) are visualized and quantified. The plots report the PSI, percent-spliced-in. *p-value < 0.05, **p-value < 0.01 (Student’s unpaired t-test; *n =* 3), error bars indicate standard deviation.

The striatum, the most severely affected brain region in HD and HD model mice, exhibited the highest number of aberrantly spliced transcripts, while cortex and liver presented more subtle splicing phenotypes (Figs 1A and S1 and S1 Table). Strikingly, the alteration in the total number of mis-spliced events in the striatum strongly correlated (0.8 to 0.97 R^2^) with *Htt* CAG length (S2 Fig), with Q175 mice showing the greatest number of abnormal splicing events at all three time points analyzed (2, 6 and 10 months) (S3 Fig). Cortical mis-splicing showed some, more variable, degree of CAG correlation at 2 and 6 months (~ 0.6 to 0.9 R^2^, S2 Fig). Similarly, modest association between liver aberrant splicing and CAG was observed, mainly at 2 months of age (S2 Fig). Interestingly, a *Htt* CAG correlated increase in aberrant striatal AS was already visible at early stages (2 months), becoming more significant as the pathologic process progressed (6–10 months) (Fig 1A and S1 Table). While RI, MXE and A3SS showed some association with CAG length, especially in the striatum at 6 months of age, the SE splicing subtype showed the strongest repeat length association (see R^2^ in S3 Fig). Importantly, the overlap, between genes exhibiting AS alteration and expression differences at each time point and with any genotype tested, was negligible (1.2%) (S4 Fig), indicating that the AS alterations were not caused by concomitant transcriptional changes.

Because the striatum exhibited a more prominent alteration in aberrant AS events during the progression of the pathologic process, we then asked whether aberrant AS events were shared among a common set of striatal genes regardless of time points and genotype. As depicted by Fig 1B, the vast majority of genes with differential AS were unique to a specific genotype and time point, though involved in redundant GO terms such as ‘synapse’, ‘cell junction’, ‘transcription’ (S1 Table). However, some common genes could be found among the lines with the most expanded CAG tracts (Q140 and Q175), mainly at 6 months of age (Fig 1B). Due to the prevalence of aberrant SE events compared to other subtypes, we performed functional enrichment analysis on genes exhibiting CAG-dependent SE mis-splicing events. We analyzed the highest CAG repeat length lines (Q140, Q175) separately, while the “lower” repeat length lines (Q80, Q92, and Q111), with fewer altered isoforms, were grouped together (Fig 1C). Functional enrichment revealed that ‘transcriptional regulation’, ‘transferase’, ‘cell junction’, and ‘synapse’ were recurrent among genotypes and the most significantly enriched terms (Fig 1C and S1 Table).

Given the predominance of aberrant SE in the mouse striatum, we wanted to experimentally validate the differential exon inclusion observed using two separate lines (Q111 and Q175) in striatal samples dissected from 6 months old mice. We prioritized a list of transcripts from the AS splicing analysis involved in ‘transcriptional regulation’, ‘synapses’ processes, pathways known to be altered in HD, and showing a significant change in percent-spliced-in (│ΔPSI│ > 0.25) of alternative exons between Q20 controls and Q111 or Q175 (S1 Table). *Crem*, a cAMP-responsive element modulator [31], *Adamts6*, ADAM metallopeptidase with thrombospondin type 1 motif, 6 [32] and *Trpm4*, TRP melastatin subgroup 4 protein—TRPM4 [33] were interesting targets, already implicated with HD and showing aberrant SE (SE increase in *Htt* CAG expanded alleles) in both Q111 and Q175 striata. Replicate RT-PCR experiments, using an independent set of Q20, Q111 and Q175 mouse striatal samples, confirmed the altered SE events (Fig 1D).

### Aberrant linear alternative splicing correlates with *Htt* CAG size in murine neural progenitor cells

In order to follow how linear AS changed during the transition from pluripotency to neuronal commitment in presence of the *Htt* CAG expansion mutation, we took advantage of a panel of mouse embryonic stem cells (mESCs) (Q20, Q50, Q92 and Q111) harboring different CAGs within a single *Htt* allele [34–36], closely mimicking the human HD mutations. mESC with different genotypes were pushed toward neural differentiation following a previously reported protocol [37]. Differentiated mouse neural progenitor cells (mNPC) were initially characterized by immunofluorescence and RT-qPCR, to confirm the correct reduction of pluripotency markers (i.e. *Pou5f1*, *Nanog*) and the induction of neural progenitor markers (i.e. *Nes*, *Vim*, *Msi1* and *Sox2*) (S5A and S5B Fig).

**Fig 2:**
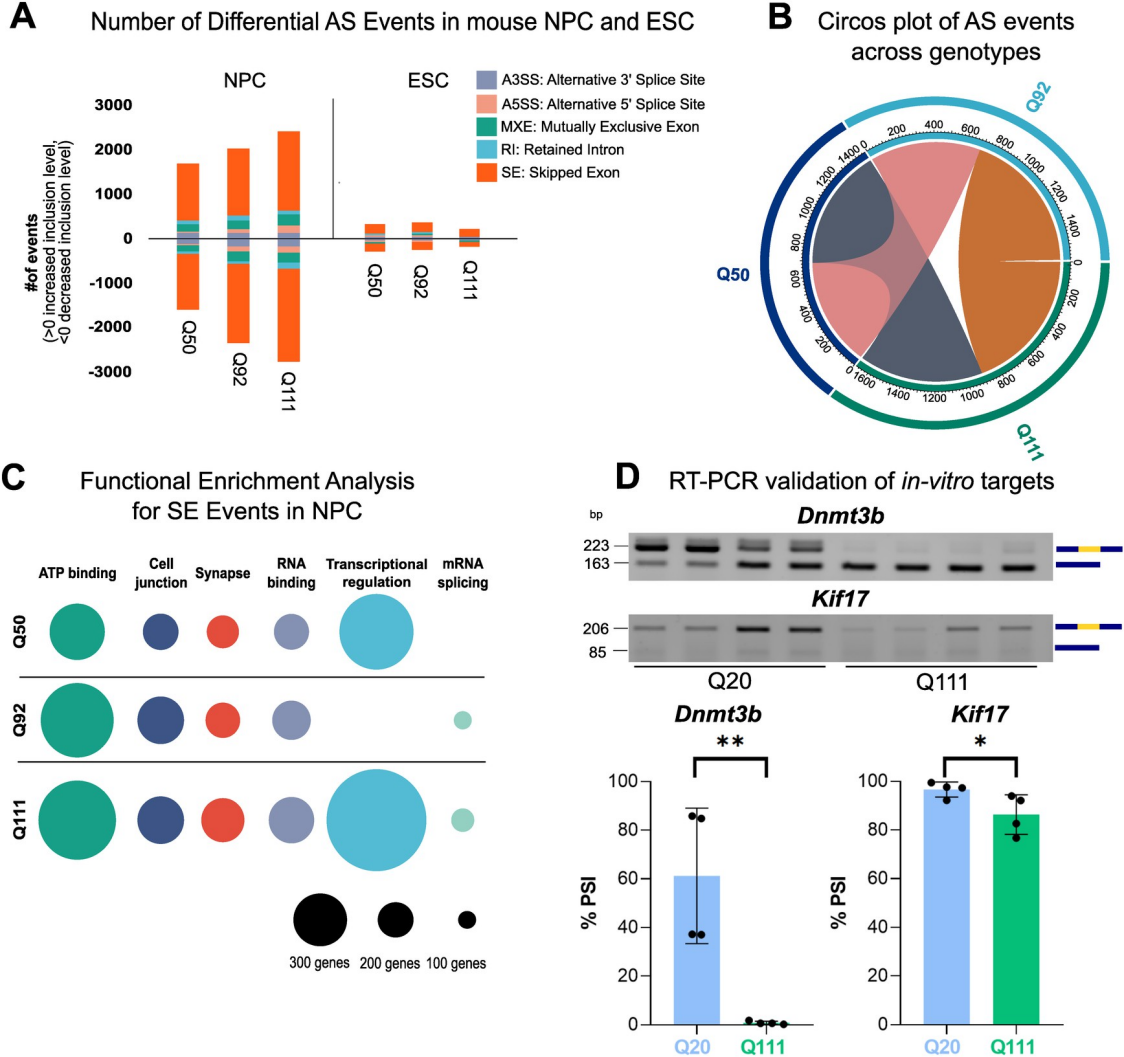
Aberrant Linear Alternative Splicing correlates with *Htt* CAG size in mNPC. **A)** The bar graph reports the number of differential AS events in the NPC and ESC from KI models of HD, presenting 3 different *Htt* CAG repeat lengths (Q50, Q92, Q111). The number of events is reported for each genotype and differentiation stage. The inclusion level is calculated in comparison to Q20 controls and the positive or negative values plotted in the graph. Further details in the Methods section. Each color of the bar chart represents a different AS type. **B)** Circos plot reports the number of transcripts in NPC—presenting differential AS events—shared between different genotypes (Q80, Q92, Q111). Conditions sharing more than 50 transcripts are depicted in colors. **C)** Weighted nodes graphical representation shows the functional enrichment analysis for transcripts presenting significant skipped exon (SE) events in NPC. Each genotype is shown separately. Nodes’ size legend is depicted at the bottom. **D)** Representative agarose gel images (top) and quantification dot-plots (bottom) report the RT-PCR results of *in vitro* AS validation for *Dnmt3b* and *Kif17*, selected transcript targets. RT-PCR assay and quantification were performed on RNA from Q20 and Q111 NPCs. *Dnmt3b* and *Kif17* isoforms presenting inclusion or exclusion of the variable exons (in yellow) are visualized and quantified. Dot plots report the PSI, percent-spliced-in. **p-value < 0.01, ***p-value < 0.001 (Student’s unpaired t-test; *n =* 4), error bars indicate standard deviation.

Following RNAseq, the mESCs and mNPCs lines were subjected to in depth transcriptional characterization for the expression of specific cell type markers (astrocytes, microglia, neurons, oligodendrocytes [38]), confirming a general expression pattern similarity among the *Htt* genotypes and a still not fully-differentiated, neuronal progenitor state (S6 Fig). Subsequently, linear AS events for mESCs and mNPCs were analyzed by the same pipeline employed for the *in vivo* analysis, comparing the different expanded *Htt* CAG repeat lengths (Q50, Q92, and Q111) to the Q20 *Htt* CAG repeat (control). Similar to the previous results (Fig 1A), a direct correlation between the total number of genes with aberrant AS events and CAG repeat length was observed in mNPCs but not in mESCs (Fig 2A). Comparable to the *in vivo* models, the highest proportion of aberrant AS events was the SE subtype and increasing *Htt* CAG size was more strongly linked to decreased rather than increased inclusion levels (Fig 2A). Importantly, also in the *in vitro* models, the AS alterations were vastly independent (79.8%) from transcriptional changes, with minor overlaps detected between differentially expressed (DE) and AS-affected genes among genotypes and differentiation time points (S2 Table). Consistent with a more homogeneous cellular population, mNPCs showed a clear overlap between genes affected by aberrant splicing (any subtype) across different genotypes (Fig 2B). Moreover, functional enrichment of genes exhibiting mis-splicing events showed that ‘RNA-binding’, ‘mRNA processing’, ‘splicing’, ‘kinase’ and ‘synapse’ were the most significantly enriched terms (S2 Table). Due to the prevalence of the SE subtype among mis-splicing events, a dedicated functional enrichment analysis was performed for each genotype separately. The analysis highlighted terms such as ‘transcriptional regulation’, ‘ATP binding’, ‘cell junction’, ‘RNA binding’ and ‘synapse’ as recurrent among the genotypes (Fig 2C and S2 Table) and mirroring the *in vivo* data (Fig 1C). Similarly, we prioritized for RT-PCR experimental validation those transcripts involved in the indicated biological pathways with statistically significant SE events with │ΔPSI │ > 0.5 between Q20 and Q111 NPC. *Dnmt3b* (SE (Q111_JCEC) ΔPSI = -0.86, SE increase in *Htt* CAG expanded alleles), involved in maintenance of DNA methylation and transcriptional regulation processes [39] and *Kif17* (Kinesin Family Member 17) (SE (Q111_JCEC) ΔPSI = -1.0, SE increase in *Htt* CAG expanded alleles) with ATPase and microtubule motor activities [40] were selected. Replicate RT-PCR experiments validated the altered SE events (Fig 2D).

### *Htt* CAG size dictates a specific linear AS signature in neural cells

**Fig 3:**
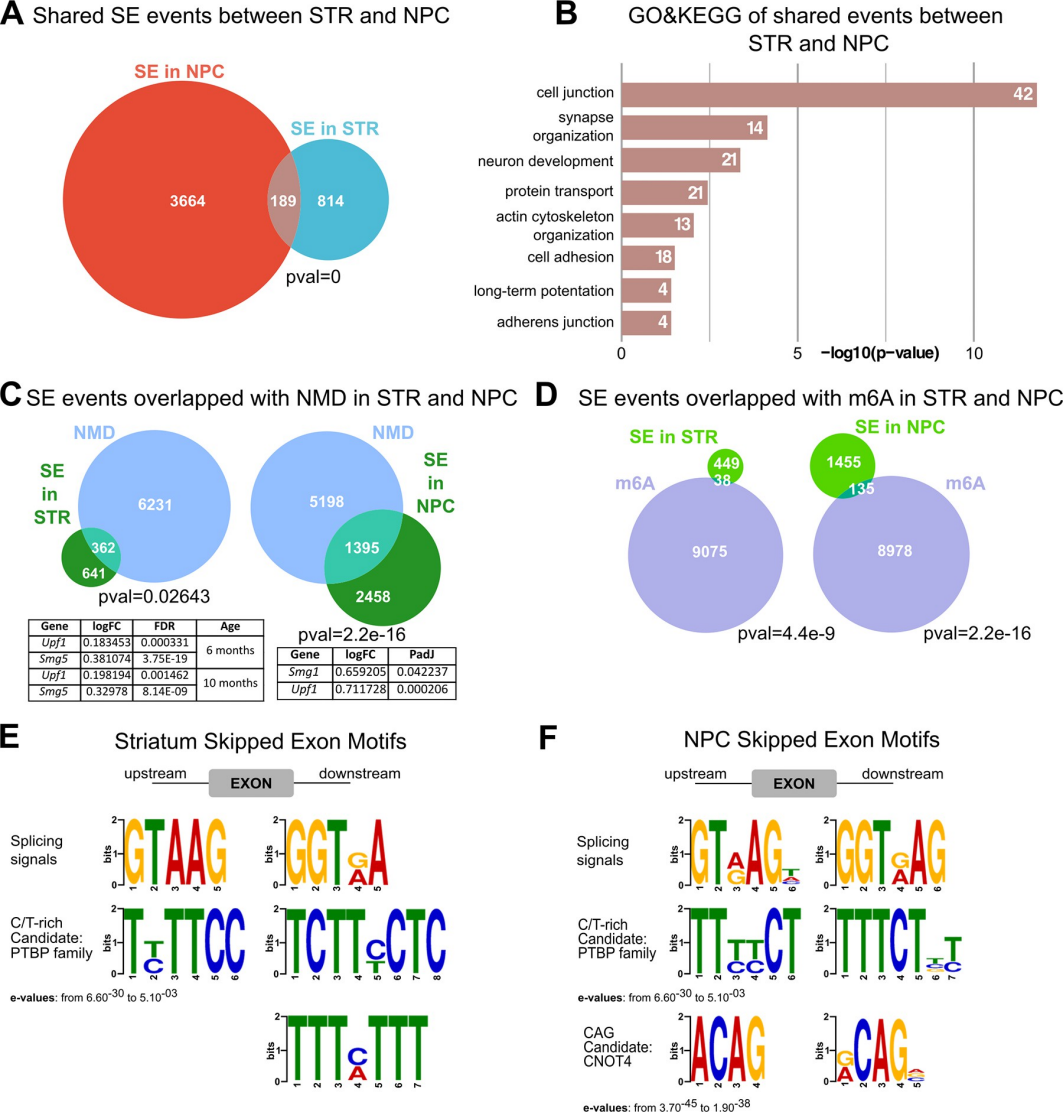
*Htt* CAG size dictates a specific linear AS signature in neural cells partly leading to NMD. **A)** The Venn diagram reports the comparison between striatal (STR) and neuronal progenitors (NPC) SE events. All genotypes (Q20, Q80, Q92, Q111, Q140 and Q175) and time points (2, 6 and 10 months) for striatal districts and all genotypes (Q20, Q50, Q92, Q111) for NPC were combined. Shared SE events (18,85% of the total striatal SE events, p-value: 0, Fisher’s exact test) are indicated in the intersection. **B)** The histogram reveals shared GO&KEGG pathway between SE and NPC events, mainly involved in ‘cell junction’ and ‘synapse organization’. Terms are ordered by −log10(p−value). The number of genes in each term/pathway is indicated (numbers within columns). **C)** The Venn diagrams show the proportion of SE events in the striatum (STR) and neuronal progenitors (NPC) also annotated as NMD. More than one third of SE events (36.1% in STR and 36.2% in NPC) is annotated as NMD. The p-value of enrichment for each intersection is indicated. The tables for striatum and NPC, highlight the transcriptional dysregulation of *Upf1*, and *Smg1*, important regulators of the NMD pathway. **D)** A smaller proportion of SE events (7.8% in STR and 8.4% in NPC) intersected with transcripts subjected to m6A RNA modification (see methods). **E-F)** Motif analysis identified the splicing factors and/or RBP binding sites in the ± 100bp upstream and downstream adjacent regions to the alternatively spliced exons for the striatum (E) and for NPC (F). The p-value of enrichment testing for individual motifs in each data set is indicated. The candidate binding splicing factor and/or RBP family is shown.

Because of the similarities in the AS results between the *in vivo* and *in vitro* HD models, we compared the genes and the events exhibiting aberrant AS in the mouse striatum and mNPCs. Genes affected by different AS event’ types are largely different: consistently, the majority of SE events in the striatum were age and genotype-specific (S7 Fig) with few genes presenting multiple AS events. Some more overlap was observed at 6 and 10 months of age (123 genes SE event, 13% of total, S7 Fig). Importantly, however, *Htt* CAG expansion caused a specific splicing disruption in a common set of SE events in the *in vivo* and *in vitro* HD models (18.85% of the total striatal SE events, p-value: 0, Fisher’s exact test) (Fig 3A), which were mainly involved in ‘cell junction’ and ‘organization of synapse’ enriched GO terms (Fig 3B and S3 Table).

Strikingly, a very similar proportion of SE events (36.1% in STR and 36.2% in NPC) was annotated as NMD, suggesting that aberrant AS *in vivo* and *in vitro* might correlate with the generation of dysfunctional transcripts, targeted to degradation. Supporting this notion, important regulators of the NMD pathway (*Upf1*, *Smg5*) were significantly transcriptionally altered in presence of the expanded mutant alleles, both *in vivo* and *in vitro* (Figs 3C and S1 and S2 Table). In search for possible splicing regulators, likely implicated in the *in vivo* and *in vitro* detected phenotypes, we resourced to GO terms and pathways analysis of transcriptionally dysregulated genes (mouse NPC S2 Table). Indeed, recurrent pathways across *Htt* CAG expanded alleles, highlighted terms correlated ‘RNA methylation’, ‘RNA N6-methyladenosine methyltransferase’. In fact, although less pervasive than NMD, also the m6A RNA modification revealed to be significantly enriched among the regions affected by aberrant SE events with similar proportion in STR (7.8%) and in NPC (8.4%) (Fig 3D). Importantly, by inspecting the sequences adjacent to the differentially skipped exons (+/-100bp) in both mouse striatum and mNPCs, we detected a specific and significant enrichment for C/T rich motifs (p-values from 6.60^-30 to 5.10^-03), typically bound by polypyrimidine tract binding proteins (PTBP) [41](Fig 3E and 3F and S3 Table). Furthermore, a shorter, CAG-rich motif was found among NPC SE motifs (p-values from 3.70^-45 to 1.90^-38), which could possibly be bound by CNOT4 [42].

### *Htt* CAG mutation impacts circRNA biogenesis in mouse neural progenitors

Considering that AS is fundamental to the biogenesis of circRNAs, we asked whether the *Htt* CAG expansion could be also associated with defective back-splicing in the *in vitro* system. First, the analysis of the percentage of circRNAs originating from each chromosome generally complied with the expected ratio relative to transcript mass in both mESCs and mNPCs (S8A and S8B Fig). CircRNA reads (average reads/sample = 3825) were then quantified in all samples by means of the DCC platform [43] (S4 Table). As shown in S9A Fig, we observed, for all genotypes, a higher number of expressed circRNAs in mNPC than in mESC samples with a corresponding higher circRNA fraction of total transcript mass (circRNA reads/circRNA + host transcript reads, S9B Fig). This trend was specific for circRNAs, given that when evaluating small RNAs [miRNAs, mitochondrial transfer RNAs (Mt-tRNAs), processed pseudogenes, ribosomal RNAs (rRNAs), small nucleolar RNAs (snRNAs), small Cajal body-specific RNAs (scaRNAs), To be Experimentally Confirmed (TEC), transcribed processed pseudogenes] (average aligned reads/sample = 1.04M, see methods), we observed a general reduction in mNPCs compared to mESCs (S10A Fig). Specifically, miRNAs and processed pseudogenes subtypes appeared to be strongly affected by neural differentiation, considerably reduced in mNPCs (S10A and S10B Fig).

While more than half of circRNA species derived from coding sequences (CDSs) and 5’-untranslated regions (5’-UTRs) in both mESCs and in mNPCs (S9C Fig), their spliced lengths were significantly longer in mNPCs (S9D Fig).

**Fig 4:**
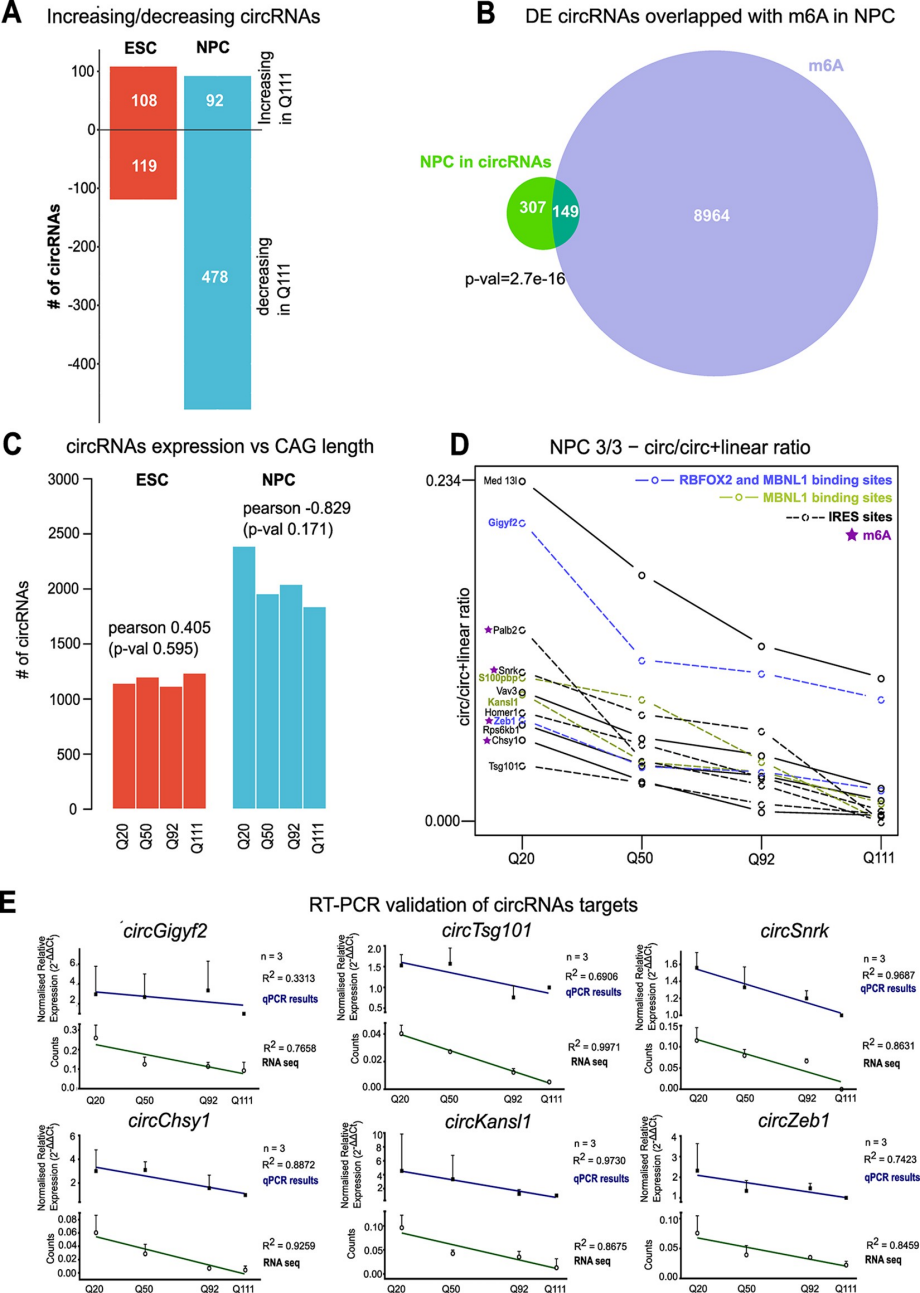
*Htt* CAG dependent reduction of circRNAs in NPC. **A)** The bar graph reports the number of circRNAs differentially expressed between *Htt* Q111 versus Q20 genotypes. The comparison is presented for pluripotent (ESC) and neural committed progenitors (NPC). The number of circRNAs increasing (*Increasing in Q111*—upper part of the plot), and decreasing their expression in Q111 versus Q20 (*Decreasing in Q111*—lower part of the plot) is depicted. **B)** The Venn diagrams depicts the proportion (32,7%) of dysregulated (DE) cirRNAs in NPCs which are also targets of m6A modification. p-value = 2.7e-16, Fisher’s exact test, associated to the intersection, is presented. **C)** The bar chart reports the total number of circRNAs expressed at each differentiation stage (ESC and NPC) and for each *Htt* CAG genotypes (Q20, Q50, Q92 and Q111). A negative correlation between the number of expressed circRNAs and *Htt* CAG length is observed (although not nominally significant) in NPC, but not in mESC cells. Pearson’s correlation R = -0.829, p-value = 0.171. **D)** The line plot describes the expression pattern of the 12 circRNAs of NPC selected by three criteria (decreasing expression and negative correlation with *Htt* CAG, significantly different expression by circTest, see Methods for further details). *Htt* CAG expansion alleles (Q20, Q50, Q92 and Q111) are presented. Of these 12 cirRNAs, 8 were predicted to have an IRES site (dashed lines), 4 were targets of m6A modification (purple stars), 2 presented MBNL1 binding sites (green lines) and 2 displayed both MBNL1 and RBFOX2 binding sites (blue lines). **E)** The line charts report the ratio (circ/linear) of the expression levels for the selected circRNAs candidates in NPC across different *Htt* CAG genotypes. RT-qPCRs results of 3 biological replicates are plotted as normalized circ/linear expression (2^^ΔΔCT^) and average ± standard deviation (SD) is presented. *Htt*Q111 genotype–with lower circ/linear ratios—was used as relativizing condition. The linear trend lines are presented for the qPCR validation (blue line) and the corresponding RNAseq data (green line) (qPCR, filled symbols; RNAseq, empty symbols). RNAseq data are presented as normalized transcript counts (counts). Pearson’s correlation R square values are indicated for the two regressions (qPCR and RNAseq).

We then analyzed the changes in circRNA expression between samples of increasing CAG length. As shown in Fig 4A, while in mESC the number of increasing and decreasing circRNAs was almost equal, circRNA expression in mNPCs predominantly decreased in Q111 vs Q20 samples (92 increasing vs 478 decreasing), while small RNA levels remained mostly unchanged (S10C Fig). Genes harboring differentially expressed circRNAs were over-represented in pathways and functions such as ‘cGMP-PKG signaling pathway’, ‘Lysosome’ and ‘DNA binding’ (S4 Table). As the presence of m6A might have implications for circRNAs biogenesis, we sought to intersect the coordinate of the DE circRNAs (+/-100bp of the circularization point) with the ones derived by m6A CLIP on mouse NPC [44]. Indeed, we uncovered a significant proportion (32.7%) of DE circRNAs also being m6A-modified (Fig 4B). Additionally, since circRNAs might host an IRES sequence, then we further investigated this hypothesis for the 570 circles, differentially regulated between Q20 and Q111. Strikingly, up to 63% (359 out of 570) of them was indeed predicted to host an IRES sequence, suggesting their possible role in polysome assembly and/or translation regulation.

To uncover a possible link between *Htt* CAG expansion and decreased circRNA expression, we counted the number of expressed circRNAs in each *Htt* CAG genotype. Although not nominally statistically significant (p-value = 0.171), possibly because of the reduced number of genotypes examined (*n =* 4), a high inverse correlation (Pearson’s coefficient = -0.829) was observed in mNPCs (Fig 4C). Such a trend was absent in mESCs. We then selected a stringently defined subset of differentially expressed circRNAs whose expression was (i) monotonically decreased with increasing CAG lengths; (ii) significantly (p < 0.05) changed according to Pearson’s correlation coefficient and (iii) significantly different as defined by CircTest algorithm [43]. 12 circRNAs fulfilling these criteria and showing a negative trend of expression with increasing *Htt* CAG length (Fig 4D), were used as circRNA candidates for experimental RT-qPCR validation. These 12 stringently defined circRNAs were resembling the general characteristics described for the entire set of 570 [8 out of 12 were predicted to host an IRES sequence; 4 out of 12 presented m6A modification sites]. Additionally, 4 out of 12 showed MBNL1 (2) or RBPFOX2 and MBNL1 (2) binding sites (Fig 4D). Biological triplicates of ribo-depleted RNAs for the 4 *Htt* CAG repeat genotypes were used for quantification of linear and circular isoforms (circRNA/circRNA+linear ratio). We experimentally confirmed the expected expression changes, i.e. circRNA expression linearly decreasing from Q20 to Q111, in 6 out of 10 targets (Fig 4E). Significantly, circ*Snrk* and circ*Kansl1*, previously characterized for their function in regulating apoptosis [45] and as miRNA sponges [46], showed the highest CAG correlation Pearson’s R^2^ and most significant p-values (circ*Snrk* R^2^ = 0.9687, p-value = 0.0313 and circ *Kansl1* R^2^ = 0.9730, *p-value =* 0.0270) (Fig 4E).

### Linear and back-splicing alterations correlate with mis-regulation of RBPs and splicing factors

**Fig 5:**
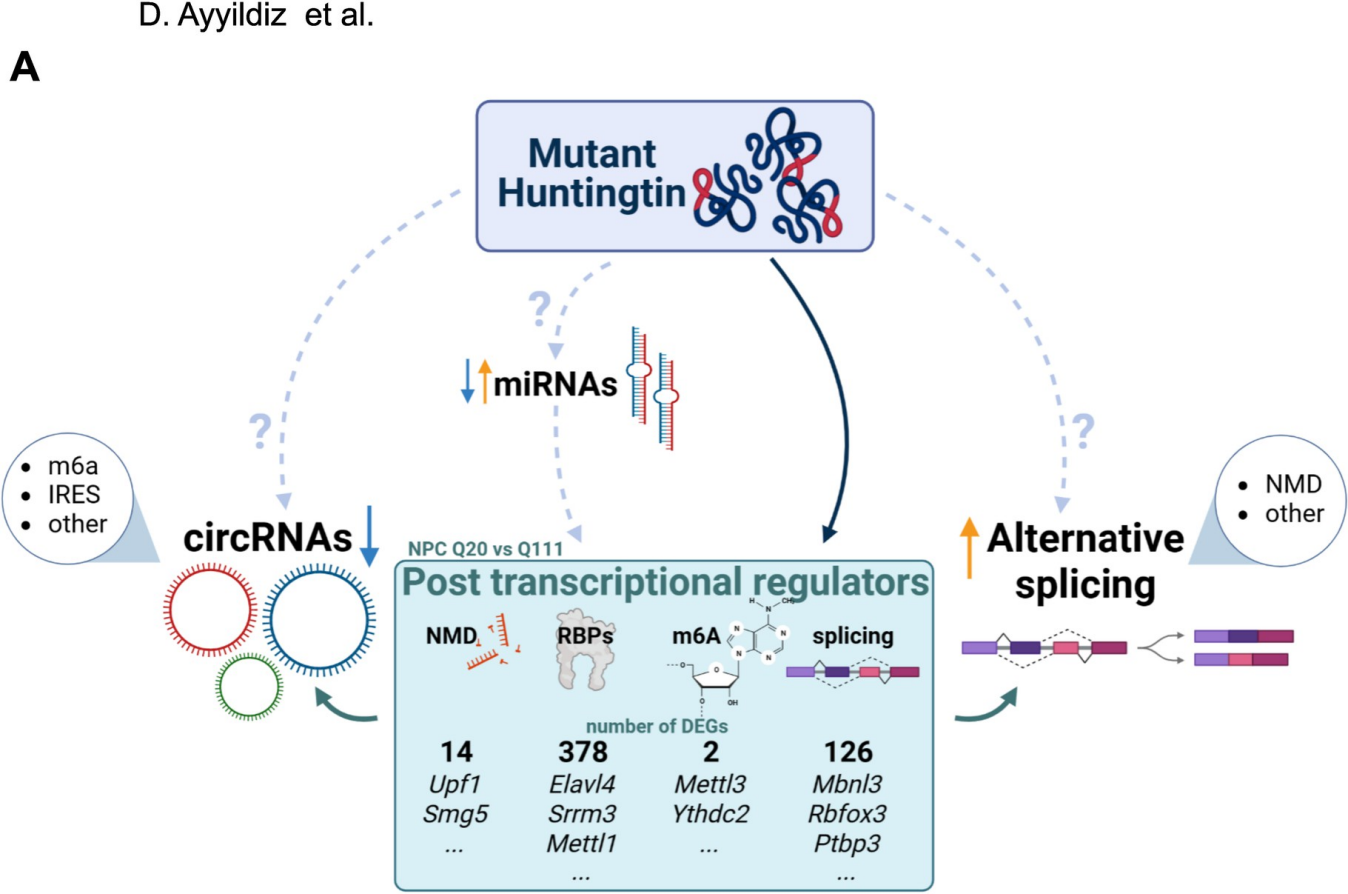
Linear and back-splicing alterations correlate with mis-regulation of post-transcriptional regulators. The schematic (created using Biorender.com) presents a possible model of action of mutant huntingtin, affecting directly or indirectly (through miRNAs) the expression of post-transcriptional regulators, eventually leading to increased alternative linear splicing and decreased circRNAs biogenesis. By comparing *Htt* Q111 versus Q20 genotypes *in vitro*, we detected 410 dysregulated post-transcriptional regulators—including RNA binding proteins (RBPs), splicing factors (splicing), Nonsense Mediated Decay factors (NMDs), and m6A players (m6A)—a proportion of which being targeted by miRNAs. Possible direct interactions between mutant huntingtin and back/linear splicing regulators might as well contribute to the described phenotypes.

In order to deepen our understanding of the effects of *Htt* CAG expansion on linear and back-splicing regulation and to gather new mechanistic insights, we initially evaluated a transcriptional effect by mutant huntingtin on RBPs and splicing factors, NMD players and writers/readers or erasers of m6A modifications. Indeed, we identified in NPC a large set (410) of post-trascriptional regulators—some of them directly interacting with wild-type and mutant huntingtin proteins [47]–DE among expanded Q111 CAG repeats and controls. Among the other, we found *Smg1*, *Upf1* implicated in NMD and *Mettl3/Ythdf2* reader and writer of m6A modification (Fig 5 and S5 Table). While a direct transcriptional regulation by mutant huntingtin cannot be ruled out, one other possible mechanism implies the dysregulation of miRNAs, which are very sensitive to neural differentiation (S10A and S10B Fig) and reported to control the stability of RNA-binding proteins and splicing factors in neurons [48–50]. Although miRNA expression strongly decreased following neural differentiation (S10A–S10C Fig), we asked whether we could identify differentially expressed miRNAs affected by the expression of *Htt* CAG expansion in mNPC. Thus, we compared Q20 versus Q111 mNPCs and identified 9 significantly differentially expressed miRNAs (S5 Table). By exploiting the mirDB database [51], we inferred their mRNA targets and intersected them with mouse splicing factors and RNA-binding proteins (RBPs) lists (see methods and S5 Table). We identified 73 transcripts (Fig 5), known RNA-binding proteins and splicing factors (of the Celf, Elav, hnRNPs, Mbnl, Ptbp, Srsf families and m6A reader Mettl3) and already shown to play a role in HD [47]. These were targets of miRNAs dysregulated by mutant huntingtin and showing expression levels changes with │Log2FC│ > 0.5 and a significant p-value (*p* < 0.05) (Fig 5). Importantly, significant overlap and enrichment was also identified between miRNA targets and transcripts showing differential alternative splicing or back-splicing in mNPC (S11A and S11B Fig and S5 Table), suggesting a possible role for miRNAs in the regulation of AS and back-splicing events [52,53]. *Upf1*, key NMD regulator, found implicated in the aberrant splicing phenotype and *Mettl3*, crucial reader of m6A RNA modification, enriched on DE circRNAs, were also validated to be transcriptionally upregulated by *Htt* CAG repeat expansion (S12 Fig). Moreover, *Ptbp3*, expressed in thymus, lymph nodes, digestive system [54], but also in adult mouse brain, was confirmed as significantly transcriptionally upregulated by qPCR experiments (S12 Fig and S5 Table). Interestingly, binding motifs, generally recognized by different Ptbps, were significantly enriched in the ± 100bp adjacent to the differentially skipped exons in both the *in vivo* and *in vitro* systems (Fig 3E and 3F), suggesting a possible role for these splicing regulators in the modulation of HD-driven aberrant RNA processing. Considering the crossregulation and functional redundancy [55] between Ptbp1/2 and 3, we decided to explore whether in presence of mutant huntingtin (Q111) the levels of Ptbps protein expression could be altered. While Ptbp3 protein showed an opposite trend (toward down-regulation in Q111), compared to what detected at the RNA level, we also unveiled significant changes in Ptbp1 and Ptbp2 abundance, with Ptbp1 in line with Ptbp3 reduction in mutant Q111 samples, while Ptbp2 showing the expected opposite drift (upregulated in mutant) (S12B Fig).

Taken together, our data suggest that mutant huntingtin might, directly or indirectly affect RBPs, splicing factors also implicated in NMD and m6A. We identified a pool of dysregulated miRNAs, sensitive to the *Htt* mutation and possibly targeting linear and back-splicing factors and validated the dysregulation of some of the post-transcriptional regulators identified. Thus, our data support a complex direct and indirect mode of mutant huntingtin regulation of canonical linear and circular RNA splicing (Fig 5).

## Discussion

Alterations in the choice of splice sites resulting in proteins with different structures and functions, altered mRNA localization, translation or decay is crucial for the complexity of the mammalian nervous system [56–59]. Splicing defects impacting the functionality of mature neurons are increasingly implicated in neurological and neurodegenerative diseases. Thus, there is a growing need to better understand these regulatory processes.

Since the discovery of the causal genetic mutation underlying HD, great efforts have been made to uncover the functions of both wild-type and mutant huntingtin, which is now thought to act in a truly pleiotropic manner. The challenge nowadays is to reveal which of these pathways might exert a crucial early role in the onset and progression of HD pathology.

Here, we investigated how RNA processing and, specifically, alternative splicing is affected by mutant huntingtin. By resourcing to publicly available RNA-seq data from *in vivo* HD KI mice (Q20, Q80, Q92, Q111, Q140 and Q175) with different CAG lengths [28] and our newly generated RNA-seq from an isogenic panel of mouse ESCs and NPCs, we uncovered a neural specific, CAG-length dependent alteration in alternative splicing. Exon skipping and, to a lesser extent, intron retention events were primarily altered. Notably, the increased total number of AS events was overt and significantly correlated with CAG length (and to a lesser extent with age) in the striatum, but less obvious within the cortex and liver, hence supporting preferential striatal vulnerability. Interestingly, significant changes in the AS pattern could already be detected prior to overt behavioral phenotypes stages (2 months). While previous reports correlated mutant huntingtin expression to mis-splicing events, locally affecting the *Htt* locus [11,60,61], and also more generally altering the whole brain’s transcriptome [14,62], this is the first evidence of a direct correlation between the number of *Htt* CAG repeats and the degree of AS, extending the repertoire of molecular phenotypes directly linked to the CAG expansion. The concept of CAG-dependency is currently extensively investigated to gain insights into how *Htt* CAG repeat length might modify HD pathogenesis [28,63,64]. Notably, our findings supporting a primary CAG length-dependent effect in striatum–with less involvement of cortex [and liver]–is well in line with previous observations that highlighted CAG length-dependent modules of co-expressed genes [28].

Similarly, the results obtained from the AS analyses, on the isogenic *in vitro* KI system [34,35,63,65,66] in the transition from pluripotency to neural committed progenitors, relevant models to study HD early phenotypes, provided an independent, genome-wide validation of the AS *Htt* CAG correlation. We confirmed an increase of aberrant AS and SE events with increasing CAG length. However, this phenotype was limited to mNPCs, which also exhibited a greater number of total AS events relative to mESCs. These observations globally confirm that neural progenitors/neural districts activate prominent neural splicing processes [67]. Genes affected by different AS event’ types *in vivo* are largely different, with limited overlap detected in the mNPCs. Additionally, very few genes present multiple AS events. In turn, this suggests that (i) the AS alteration correlation with CAG length likely requires factors expressed in the neural lineage and (ii) the process seems to be subjected to a complex regulatory scheme, possibly driven by altered expression levels, localization, and interaction of post-transcriptional regulators with cell type specificity and variances with disease’s progression. Consistently, limited overlap was found between genes affected by aberrant AS in early studies from HD *post mortem* brains [14], while a more significant intersection emerged by comparing to human and mouse datasets from HD by Elorza et al., 2021 [62]. Functional enrichment analyses for transcripts experiencing SE events, *in vivo* and *in vitro*, revealed GO and pathways already associated with HD [68–70], with prevalence of adhesion and neural-related functions. Taken together, these findings suggest that linear splicing becomes progressively dysregulated as CAG length increases, specifically in neuronally-committed cells and adult striatum, thus possibly contributing to HD pathogenesis.

We next searched for a glimpse of the possible molecular mechanisms involved in the process. Since altered AS might, in some cases, can be propaedeutic to NMD, we explored this hypothesis by overlapping our CAG-expansion driven SE events in striatum and mNPC with the ones annotated for NMD. We indeed found a significant portion (36%) of the SE events intersecting with the ones leading to NMD. This proportion was comparable in both the *in vivo* and *in vitro* systems. Importantly, some of the crucial NMD regulators (*Upf1*, *Smg1*) were transcriptionally dysregulated following mutant huntingtin expression, thus suggesting a direct/indirect mode of mutant protein to activate NMD. Consequently, we sought to explore whether *Htt* transcript expression levels could be correlated with the observed phenotype. Though the expected [71,72] significant reduction in *Htt* expression could be observed with higher expansion genotypes (especially Q140 and Q175) and a milder reduction was associated with aged mice, this trend was identical in cortex, striatum, but also liver districts, minorly affected in their AS regulation. Thus, *Htt* expression doesn’t seem to play a clear role in the regulation of these phenotypes.

AS regulation is also crucial to the biogenesis of circRNAs, stable, neuronally-enriched, circular non-coding RNAs [21,73], with important roles during neural development [74,75] and in brain function [24,76–78]. While their contribution to brain pathologies and specifically to neurodegenerative diseases is becoming recognized [77–79], circRNAs remain largely neglected in HD. We aimed to provide a first characterization of back-splicing regulation in the presence of mutant huntingtin expression, exploiting the isogenic *in vitro* KI system. In contrast to what was observed for linear splicing, we detected a general decrease in circRNAs abundance with increasing CAG length. While the impact of mutant huntingtin on circRNAs biogenesis is remarkable when comparing the two extreme genotypes (Q20 versus Q111), the genes affected by AS and back-splicing are largely different, and also enriched in different GO and pathways terms. However, once again, the more striking phenotype appeared to be confined to neural progenitors with minimal changes in mESCs. It is unclear, however, whether these circRNAs play known or novel regulatory roles [80], and whether their depletion might contribute to HD pathogenesis.

Taken together, our observations demonstrate an anticorrelation of linear AS and back-splicing with the number of CAG repeats, thus suggesting a possible link between the two splicing types. Previous studies reported exon skipping as a promoter of skipped-exon circularization [20]. However, other data suggested that alternative splicing is likely in competition with back-splicing [19,73]. Our results support the latter idea, where a global increase of SE events downregulates back-splicing efficiency although it remains unclear whether the *Htt* CAG mutation primarily affect linear splicing, exon circularization, or both simultaneously. Importantly, as previously indicated [22], the m6A RNA modification appears to be associated with exons circularization—but not so significantly with SE events on linear transcripts. Indeed, more than 30% of the differentially expressed circRNAs sequences were also targeted by the m6A machinery. Importantly, we explored the GO terms and pathways enriched for the genes differentially expressed between the two extremes genotypes in mNPC (Q20 vs Q111), specifically searching for possible RBP or splicing regulators that might be involved in the backsplicing phenotypes. Among the others, terms associated to ‘RNA methylation’, ‘methylation’, ‘RNA modification’ stood out as most significant. In fact, different writers/readers of the m6A pathways resulted to be differentially expressed (*Mettl3/Mettl14*, *Ythdf2*, *Igf2bp1/2/3*), thus suggesting a regulatory role of mutant huntingtin in this process. However, dedicated functional experiments will be required to fully understand the impact of m6A modification in the regulation of splicing and back-splicing events by mutant huntingtin.

The effect exerted by the *Htt* CAG expansion on circRNA biogenesis in mNPCs might be exacerbated by the higher number of circRNAs detected at this developmental stage, already reported in a number of studies [75,81,82]. On the other hand, it might suggest that the *Htt* CAG expansion could, directly or indirectly, regulate some neural splicing factors also controlling back-splicing. The *Htt* CAG expansion, thus, might induce an abnormal interaction and/or alter the expression levels of small RNAs, splicing factors or RBP, specifically in neural-lineages [14], in turn affecting the two types of splicing (S5 Fig).

While the transcriptional effects on different splicing regulators can be directly ascribable to mutant huntingtin nuclear function, other indirect processes might also be involved. Here, we explored a network of miRNAs and their targets to expose mechanistic insights. miRNAs revealed to be altered by neural differentiation and, to a lesser extent, by the *Htt* CAG expansion. Interestingly, a fraction of the targets of mutant huntingtin’s-dysregulated miRNAs, were significantly enriched for RBP and splicing factors, with members of the Ptbp, Celf, Srsf, NMD, m6A writers/readers and hnRNPs families, also showing transcriptional and protein alterations, validated in independent systems (Figs 5, S11, and S12 and S5 Table), but still not fully dissected from the molecular point of view. This suggests that AS regulatory elements might be directly or indirectly (via miRNAs alteration) dysregulated by *Htt* CAG expansion (Fig 5).

In conclusion, we identified splicing and back-splicing as highly impacted molecular processes in HD model systems. Although previous reports associated mutant huntingtin expression to linear splicing dysregulation [14,60,61], this is the first study reporting the relationship between magnitude of AS alterations and *Htt* CAG length and the very first evidence of circRNA deregulation in HD. Moreover, the identification of aberrantly regulated NMD players, m6A writers and readers, RBPs, and splicing factors, specifically in neural committed cells and in combination with *Htt* mutation, proposes novel molecular players contributing to HD pathogenesis and delineating new targets of therapeutic intervention.

## Materials and methods

### Ethics statement

All animal experiments were conducted to minimize pain and discomfort, under approved Institutional Animal Care and Use Committee (IACUC) protocol of the Italian Ministry of Health (project authorization n. 781/2016-PR) and the Massachusetts General Hospital.

### Generation and characterization of mNPC with different *Htt* CAG sizes

The isogenic panel of wild-type and heterozygous *Htt* CAG knock-in *Htt*^Q20^, *Htt*^Q50^, *Htt*^Q92^ and *Htt*^Q111^ mESCs, kindly provided by Dr. Marcy E. MacDonald (Massachusetts General Hospital and Harvard Medical School, Boston, USA), were cultured as described previously [34,35,65]. Pluripotent cells were maintained in KnockOut DMEM (Gibco), supplemented with 15% of ESC-grade FBS (Gibco), 2 mM L-glutamine (Gibco), 100 U/ml Penicillin/Streptomycin (Gibco), 1% Non-essential Amino Acids (Gibco), 0.1 mM 2-mercaptoethanol (Sigma) and 1000 U/ml of leukemia inhibitory factor (LIF) (Voden), on plates coated with 0.1% gelatin (Millipore) or on a feeder layer of CF-1 IRR mouse embryonic fibroblast (TebuBio).

Neural differentiation was performed as previously described [37]. Briefly, self-renewing mESCs were dissociated and plated onto 0.1% gelatin-coated plates at a density of 0.5–1.5 10^4^cells/cm^2^ in N2-B27 medium. After 7 days, cells were detached using Accutase (Thermo Fisher Scientific) and plated on 3 μg/ml laminin-coated dishes in neural stem (NS) expansion medium [composed by Euromed-N (Euroclone) supplemented with 20ng/ml FGF (R&D) and EGF (Sigma), 1% N-2 Supplement (Gibco), 2 mM L-glutamine (Gibco) and 100 U/ml Penicillin/Streptomycin (Gibco)] [37]. Mouse neural progenitor cells were routinely passaged 1:2–1:4 every 3–5 days using Accutase and maintained in NS expansion medium on laminin-coated plates (Sigma, 3 μg/ml). Both mESCs and mNPCs were incubated at 37°C and 5% CO_2_. The pluripotency of mESCs and their differentiation to mNPCs was evaluated by RT-qPCR using stage specific markers (full list of primers used in S6 Table).

### Immunofluorescence

Cells were seeded on a 24-well the day before the experiment and then fixed with 4% PFA for 15 minutes at room temperature, permeabilized with 0.5% Triton X-100 in 1X PBS and incubated with blocking solution (0.3% Triton X-100, 5% FBS in 1X PBS) for 1 hour. Primary antibody incubation was carried out at 4°C overnight, followed by three washes with 1X PBS. Proper secondary antibodies were eventually used and images acquired using a confocal microscope (Leica TCS SP5). The following antibodies were employed: rat anti-Nestin (Santa Cruz Biotechnology), rabbit anti-Sox2 (GeneTex), goat Alexa Fluor 546 and 647 (Life Technologies). Nuclei were stained using Hoechst 33342 (Life Technologies), diluted 1:20000 in 1X PBS.

### Tissues dissection and isolation

3 *Htt* knock-in mouse lines with an *Htt* CAG repeat knock-in allele (*Htt*^Q20^, *Htt*^Q111^ and zQ175) (C57BL/6 J inbred background) have been described previously [35,83,84]. Mice were maintained as heterozygotes and genotyped according to previously published protocols [35,83,84].

Mice were sacrificed by CO_2_ asphyxiation followed by cervical dislocation. The brain regions of interest (striatum and cortex) were dissected on ice, rapidly removed, snap frozen and stored at −80°C for further use. Each group/genotype included equal number of males and females to avoid sex-dependent bias, was collected at 6 months of age.

### RNA extraction and reverse transcription

Isolation of total RNA from tissues and cells was performed using TRIzol-based extraction method according to the manufacturer’s protocol (Thermo Scientific). RNA was resuspended in nuclease-free water and quantified by a Nanodrop 2000 spectrophotometer (Thermo Scientific). The quality of RNA was estimated by using the RNA 6000 Nano or Pico Bioanalyzer 2100 Assay (Agilent). RNA samples with RNA integrity number between 6.8 and 9.5 were used. Unless otherwise noted, 1 μg of total RNA was reverse transcribed using SensiFAST cDNA Synthesis Kit following the manufacturer’s protocol (Bioline).

### Primer design

Transcripts were searched on Ensembl genome browser 95 (https://www.ensembl.org/Mus_musculus/Info/Index) and primers were designed on the coding sequence of each gene. Primers for end-point PCR were generated using the default setting on Primer3web (http://primer3.ut.ee/) and those for quantitative PCR by using Roche Universal ProbeLibrary for Mouse (https://lifescience.roche.com/) and Primer-BLAST NIH tool (https://www.ncbi.nlm.nih.gov/tools/primer-blast/). The list of primers used in this study is presented in S6 Table.

### Quantitative PCR analysis

Quantitative polymerase chain reaction (qPCR) was performed using SensiFAST SYBR No-ROX Kit (Bioline, BIO-98020) according to the manufacturer’s instructions. Data was normalized with RNA Polymerase II subunit A (*Polr2a*) as housekeeping gene (ΔCT) and analyzed with the 40-ΔCT method. The 40-ΔCT method was used to distinguish between down-regulation and overexpression of markers of mESCs and mNPCs.

### Validation of linear AS candidates’ transcripts

Candidate transcripts presenting differential linear AS among the different conditions were filtered on the basis of two parameters: (i) the percentage difference between the length of the target exon–included or excluded—and the invariant adjacent exons should be higher than 25% and (ii) the percentage of target exon inclusion/exclusion difference between *Htt*^Q111^ and *Htt*^Q20^ or *Htt*^Q175^ and *Htt*^Q20^ mice should be higher than 25%. For the selected candidates, primers were designed targeting the invariant adjacent exons to amplify the two splicing isoforms. RT-PCR was then performed using Phusion Green Hot Start II High-Fidelity PCR Master Mix (Thermo Scientific) and 20–100 ng cDNA. PCR amplicons were resolved on 2% agarose gels pre-stained with Xpert Green DNA Stain (Grisp) in 1× TBE buffer (89 mM Tris-borate and 2 mM EDTA, pH 8.3). The intensity of the bands was quantified using ImageJ software (NIH, Bethesda, MD) and the percent spliced-in value was calculated as (intensity of band with exon inclusion divided by the sum of the intensity of bands with both exon inclusion and exclusion) x 100. Replicate experiments were carried out and statistical analysis was conducted using Student’s unpaired t-test. p-value ≤ 0.05 (*) or p-value ≤ 0.01 (**).

### Validation of expression changes for selected circular RNA candidates

For circular RNA validation experiments, 10 μg of total RNA was depleted of rRNAs by using the human/mouse RiboMinus Transcriptome Isolation Kit (Invitrogen). Ribosomal RNA depletion was confirmed by an Agilent 2100 Bioanalyzer (Agilent). Ribo-depleted RNA was reverse-transcribed and quantitative PCR was performed by employing iTaq Universal SYBR Green Supermix (Biorad). To test the correlation between circular RNAs candidates and the number of CAG repeats to faithfully recapitulate the bioinformatic analysis, we considered the expression of both linear and circular isoforms of the selected transcripts. We designed convergent and divergent primer sets for each candidate (S6 Table). To specifically target the circular isoform, the backspliced junction was selected. Expression values were obtained employing the 2^^-ΔΔCT^ method, normalizing with *Polr2a* and *Actb* housekeeping genes (HKG) and relativizing to Q111. The selected HKGs were chosen among a panel of 5, showing a more stable expression in our *Htt* KI samples. PCR amplicons were loaded on a 2% agarose gel, bands were carefully excised, purified using PureLink Quick Gel Extraction and PCR Purification Combo Kit (Bioline) and eventually Sanger sequenced to finally prove their identity and the back-splice junction. GraphPad Prism (GraphPad Softwares) was used to establish a regression line between circular to linear ratios and the CAGs serie (Q20, Q50, Q89, Q111). Finally, R^2^ values and corresponding p-values were calculated.

### Validation of expression changes for selected post-transcriptional regulators

For differentially expressed post-transcriptional regulators (*Ptbp1/2/3*, *Upf1*, *Mettl3*) validation, RNA was extracted from an independent batch of Q20 and Q111 mNPCs. 1 ug of RNA for each sample was retrotranscribed (RT) using SensiFAST cDNA Synthesis Kit (Bioline), according to manufacturer’s instructions. Samples were diluted 1:10 and qPCR was performed using iTaq Universal SYBR Green Supermix (Bio-Rad), according to the manufacturer’s instructions. Data was normalized on phosphoglycerate kinase 1 (*Pgk1)* as housekeeping gene (ΔCT) and analyzed with the 2^^-ΔΔCT^ method. One tail, unpaired t-test was performed to calculated statistical significance. Three biological replicates for each genotype were analyzed and plotted with mean and standard deviation using GraphPad Prism software. Primers used are present in S6 Table.

### Western blot analysis

Proteins were extracted from mNPC lines using RIPA Lysis Buffer (Pierce) and following manufacturer’s instructions. Proteins were quantified using BCA Protein Assay kit (Pierce). 30 μg (For Ptbp3) or 15 μg (Ptbp1 and 2) were ran on 4–12% NuPAGE Bis-Tris precast gel (Pierce) in MOPS running buffer. Transfer was performed in Tris/Glycine buffer (BioRad) on Nitrocellulose membrane (Amersham) for 1h at 100V. Blocking was done in 5% milk in PBS-Tween. Antibodies [Ptbp1 (12582-1-AP,1:5000) Ptbp2 (55186-1-AP, 1:500), Ptbp3 (14027-1-AP 1:500) and Hsp90 (GTX13492, 1:5000) were diluted in blocking solution. Images were acquired using ChemiDoc Imaging System (BioRad). Protein expression was calculated by normalizing on housekeeping gene (Hsp90) and relative to the average of Q20 mNPCs. Two tails, unpaired t-test was performed to calculate statistical significance. Four biological replicates for each genotype were analyzed and plotted with mean and standard deviation using GraphPad Prism software.

### Library preparation and RNA sequencing

For total RNA, ESC and NPC (at P14 and P16 passages) samples were subjected to DNAse I treatment (Ambion) according to manufacturer instructions. Ribodepletion and barcoded stranded RNA-seq libraries preparation was performed by the European Molecular Biology Laboratory Genomic Core facility, following the standard Illumina protocols. Libraries were then sequenced with NextSeq 500 paired-end, 75bp reads, obtaining ~60M reads/library.

Similarly, miRNA libraries were generated by the European Molecular Biology Laboratory Genomic Core facility according to standard procedures and were sequenced on the Illumina HiSeq 2500 using single-end, 50bp reads obtaining ~15M reads/sample.

### RNA-seq data, miRNA-seq and circRNA data analysis

Data from RNA-seq and miRNA-seq on mESC and mNPC allelic series were processed as follows. Reads filtering (minimum base quality of Q30) and adapter trimming was performed with Trim Galore (http://www.bioinformatics.babraham.ac.uk/projects/trim_galore/)). Remaining reads were then aligned with STAR v2.5.3a [85] to the mm10 assembly of the mouse genome coupled to the M14 Gencode genes annotation [86]. STAR parameters were set as recommended by the DCC pipeline [43] to allow for the subsequent detection of circRNAs through chimeric junctions. Reads derived from circular RNA species were extracted from the Q20—Q111 samples of mESC and NPC cells by the DCC pipeline v0.4.4 [43], run with default parameters. Repeats annotation provided to DCC was obtained from the UCSC Genome Browser [87], by combining the RepeatMasker and Simple Repeats track for the mm10 mouse assembly. Reads from circRNA host genes were also quantified by DCC in the same run. Total transcript mass was computed for a given transcript as the sum of its linear and corresponding circular RNA reads count. The relative abundance of the circular form of the transcripts was then computed as circular RNA read counts / circular + linear RNA read counts.

All p-values were computed by a Wilcoxon rank-signed test unless explicitly stated. Exons/introns annotation and the number of protein-coding genes per chromosome were obtained from UCSC [87] for the mm10 mouse genome assembly. These annotations were then used to compute circRNAs spliced length and the enrichment of expressed circRNAs in chromosomes (by a Fisher test).

Gene Ontology and pathways enrichment were computed with the topGO [88] and clusterProfiler [89] R packages, using a BH adjusted p-value threshold of 0.05.

Differential expression of circRNAs was computed by the CircTest R package [43]. circRNAs were filtered by requiring at least five reads in at least one sample and at least 5% of total transcript mass in at least one condition. circRNAs having an FDR lower or equal to 0.05 were considered to be differentially expressed.

### Motif analysis

The sequence of the 100nts upstream and downstream of significant skipped exons events were extracted with the biomaRt R package [90]. A motif search was performed with DREME v4.10 [91], considering only same-strand matches, using a background sequence set generated by DREME via shuffling of input sequences and a 1.0E-03 threshold on the E-value to select significant motifs.

### Differential expression and differential linear alternative splicing analysis

For the *in vivo* differential splicing analysis, the raw data from the study of Langfelder P et al (2016) was used [28]. Striatum (GSE65774), cortex (GSE65770) and liver (GSE65772) mRNA expression profile datasets were retrieved for the analysis through the online database HDinHD portal (https://www.hdinhd.org/). At each of 3 time points (2, 6, 10 months), 8 heterozygous knock-in mice from each of the 6 *Htt* CAG repeat lengths (Q20, Q80, Q92, Q111, Q140, and Q175) were used, resulting in 48 samples from each tissue and each time point. Raw reads were subjected to sequence quality control using FastQC (http://www.bioinformatics.babraham.ac.uk/projects/fastqc/)). Removal of low-quality reads and trimming of the adapter sequences were achieved by Trim Galore (http://www.bioinformatics.babraham.ac.uk/projects/trim_galore/). In order to eliminate variability between sequencing runs the reads were trimmed to 45 bp by Trim Galore, resulting in the removal of only 4% of the reads. Raw sequences were aligned to mm10 mouse genome assembly (UCSC) with STAR RNA-seq aligner version 2.5.3a [85] using standard settings. The differential alternative splicing (AS) events between each of 5 samples for *Htt* CAG repeat lengths (Q80, Q92, Q111, Q140 and Q175) and Q20 (as control) were identified by rMATS v4.0.1 [30] (http://rnaseq-mats.sourceforge.net) that detects five major types of AS events from RNA-Seq data with replicates [29,30]. Analyses results were then further processed with R/Bioconductor.

For the *in vitro* differential splicing analysis, both mESC and mNPC samples were subjected to the same pipelines used for *in vivo* differential splicing analysis with same parameters and tools. For the analysis of the differential alternative splicing (AS) events of 3 samples for *Htt* CAG repeat lengths (Q50, Q92, and Q111), Q20 samples were used as control.

Differential expression analysis was performed on striatum samples for *in vivo* with read counts output of the alignment which was performed in previous step. EdgeR (v3.24.3) was used within the Bioconductor environment in R and p-values were adjusted for multiple comparisons using the Benjamini–Hochberg method within each contrast and genes with FDR-adjusted p-value < 0.05 were considered significantly differentially expressed. To identify the genes both differentially expressed and alternatively spliced (all the different AS subtypes were included), differential expression and alternative splicing results were overlapped.

The functional enrichment analysis of the genes with AS events was performed using the DAVID functional enrichment tool v6.8 mainly based on GO terms (biological process, cellular compartment and molecular function), KEGG pathway, InterPro and UniProtKB keywords. The enriched terms were filtered according to FDR adjusted p-value <0.05.

### Cis-elements analysis

m6A-modified sites were retrieved for NPC cells from the GSE104686 dataset [44], obtained through the REPIC database [92], and intersected with splicing events through bedtools [93,94]. IRES elements were predicted by using the BLAST function of IRESite (iresite.org) [95].

Potential binding sites for RBPs were identified in circRNAs with Biopython using CISBP-RNA motifs PWM [96] with a false positive rate threshold of 0.01 and a percent match identity of 95%. circRNAs coordinates were also intersected with binding sites for Mbnl1 (dataset GEO ID: GSM981230) and other mouse RBPs from the POSTAR database [97] with bedtools [93,94].

## Supporting information

S1 Fig Altered linear alternative splicing in the liver of KI animal models of HD. The bar graph reports the number of differential AS events in the liver from mouse KI models of HD, presenting 6 different *Htt* CAG repeat lengths (Q20, Q80, Q92, Q111, Q140 and Q175) and 3 time points (2, 6, and 10 months). The inclusion level is calculated in comparison to Q20 controls and the positive or negative values are plotted in the graph. The number of events is reported for each genotype and time point. Source data by Langfelder P. et al (2016) [28]. Further details can be found in the Methods section and S1 Table. Each color of the bar chart represents a different AS type.
(TIFF)

S2 Fig *Htt* CAG length correlation with linear alternative splicing events in KI mouse models. The bar graphs report the total number of differential AS events in the striatum (upper row, light blue), cortex (mid row, green) and liver (bottom, purple row) from mouse KI models of HD, presenting 6 different *Htt* CAG repeat lengths (Q20, Q80, Q92, Q111, Q140 and Q175) and 3 time points (2, 6, and 10 months). The inclusion level is calculated in comparison to Q20 controls and the positive or negative values are plotted in the graph. The number of events is reported for each genotype and time point (different Y-axis values are presented). Source data by Langfelder P. et al (2016) [28]. Further details can be found in the Methods section and S1 Table. The Pearson’s correlation (R^2^) between differential AS and *Htt* CAG expansion is plotted in each graph. Standard deviations and trend lines are presented.
(TIFF)

S3 Fig *Htt* CAG length correlation with different alternative splicing events’ types in the striatum of KI mouse models. The bar graphs report the number of different alternative splicing events’ types (A3SS: Alternative 3’ splice site, A5SS: Alternative 5’ splice site, MXE: Mutually exclusive exon, RI: Retained intron and SE: Skipped exon) significantly altered in the striatum of mouse KI models of HD, presenting 6 different *Htt* CAG repeat lengths (Q20, Q80, Q92, Q111, Q140 and Q175) and 3 time points (2, 6, and 10 months; Blue, Orange and Green, respectively). The inclusion level is calculated in comparison to Q20 controls and the positive or negative values are plotted in the graph. The number of events is reported for each genotype and time point. Source data by Langfelder P. et al (2016) [28]. Further details can be found in the Methods section and S1 Table. The Pearson’s correlation (R^2^) between different AS events’ types and *Htt* CAG expansion is plotted in each graph. Standard deviations and trend lines are also presented.
(TIFF)

S4 Fig Differentially expressed genes presenting AS events in the striatum tissue of KI mouse models. The bar graph presents the number of differentially expressed genes (DEG) which also are characterized by at least one significant AS event in the striatum of KI animal models of HD. Logarithmic fold change (LogFC) of their expression compared to Q20 control is reported in the y-axis. Transcripts are filtered based on significant p-value (p-value <0.05). Transcripts names and condition were the differential expression was observed are indicated in the x-axis. Transcripts are divided accordingly to the specific AS event type (A3SS: Alternative 3’ splice site, A5SS: Alternative 5’ splice site, MXE: Mutually exclusive exon, RI: Retained intron and SE: Skipped exon). Color code defines events types and DEG bars.
(TIFF)

S5 Fig *Htt* CAG-expansion mESC and mNPC exhibit similar stage-appropriate morphological and molecular characteristics. **A)** Phase contrast micrographs of heterozygous *Htt* CAG knock-in *Htt*^Q20^, *Htt*^Q50^, *Htt*^Q92^ and *Htt*^Q111^ (Q20, Q50, Q92, Q111) NPC lines derived from neural differentiation of ESC display the appropriate morphology with neurite extensions. **B)** Fluorescent images of cells, with Hoechst 33342 stained nuclei, show proper expression of Sox2 and Nestin neuroectodermal markers in the NPC for each genotype. Scale bars = 50 μm. Nestin is enlarged to better appreciate neuronal processes. Scale bars = 25 μm. **C)** Bar graphs plot the relative normalized mRNA expression levels of pluripotency marker genes *Pou5f1* and *Nanog* and neuroectodermal marker genes N*es*, *Vim* and *Msi1* as determined by RT-qPCR amplification assays. Error bars represent standard deviations from the mean of two biological and two technical replicates.
(TIFF)

S6 Fig *Htt* CAG-expansion ESC and NPC express appropriate transcriptional markers. The heatmap describes the normalized counts per millions (cpm) of cell types specific markers as described in [38]. Astrocytes, microglia, neurons, oligodendrocytes, neural progenitors and pluripotent cells were analysed for heterozygous *Htt* CAG knock-in *Htt*^Q20^, *Htt*^Q50^, *Htt*^Q92^ and *Htt*^Q111^ (Q20, Q50, Q92, Q111) in their transition from ESC to NPC. This deep transcriptional characterization confirms a similar pattern of expression between different genotypes and a still not fully committed neuronal progenitor state.
(TIFF)

S7 Fig Skipped exon (SE) events in the striatum (STR) are age and genotype specific. The upset plot displays the number of SE events shared among different time points (2, 6 and 10 months) within the mouse striatum. For each time points, all the KI mice genotypes with expanded CAG tract were pooled together. The number of events within each intersection is presented in the vertical bars. Intersection groups (lines) or single time points (dots) are shown in the lower panel. Sample set size is indicated at the bottom left of the panel.
(TIFF)

S8 Fig Distribution of circRNAs across different chromosomes in ESC and NPC. **A-B)** The bar charts display the percentage of detected circRNAs originating from each chromosome in ESC **(A)** and NPC **(B)**. Enrichment of circRNAs, using the number of protein-coding genes contained in each chromosome as a background, was computed by a Fisher test and significant p-values are displayed over the graph bars (* < 0.05, ** < 0.01, *** < 0.001).
(TIFF)

S9 Fig Neural differentiation impacts on circRNAs biogenesis. **A)** The bar chart shows the number of detected circRNAs (>1 count/sample, see also Methods), comparing pluripotent (ESC) and neural committed progenitors (NPC). Series of 4 *Htt* CAG expansion alleles (Q20, Q50, Q92 and Q111) are presented. Average circRNA count from two biological replicate experiments is plotted. **B)** Bars chart presents the circRNA fraction of total transcript mass in ESC and NPC different genotypes. **C)** The bars chart reports the percentage of circRNAs derived from specific transcripts areas: transcript coding sequence (CDS), 3’ or 5’ untranslated regions (3UTR/5UTR), whole transcript or other. The data compare ESC and NPC conditions. All *Htt* genotypes were combined. **D)** The box plot displays the spliced length distribution (in base pairs, bp) [average ± standard deviation (SD)] for circRNAs in the various conditions of ESC and NPC cells. *Htt* genotypes as in a). Wilcoxon test p-values of the difference between corresponding conditions of ESC and NPC are shown as stars (* < 0.05, ** < 0.01, *** < 0.001).
(TIFF)

S10 Fig Neuronal differentiation impact on small RNAs. **A)** The coloured bar graphs report the number of small RNAs (>1 count/sample, see also Methods) comparing ESC and NPC conditions. Series of 4 *Htt* CAG expansion alleles (Q20, Q50, Q92 and Q111) are presented. Different classes of small RNAs are examined. Abbreviations as follows: microRNAs (miRNAs), Mitochondrial transfer RNAs (Mt-tRNAs), processed pseudogenes, ribosomal RNAs (rRNAs), small nucleolar RNAs (snoRNAs), small nuclear RNAs (snRNAs) small Cajal body-specific RNAs (scaRNAs), To be Experimentally Confirmed (TEC), transcribed processed pseudogenes. **B)** The heatmap presents the number of expressed (> 1 count per million, CPM) small RNAs among heterozygous *Htt* CAG knock-in *Htt*^Q20^, *Htt*^Q50^, *Htt*^Q92^ and *Htt*^Q111^ (Q20, Q50, Q92, Q111), comparing ESC and NPC lines. Different classes of small RNAs are examined. Abbreviations as in GENCODE transcript biotypes [86]. Color code bar (upper right) reports the number of expressed small RNAs in each condition. **C)** The bar chart shows the number of small RNAs differentially expressed between *Htt* Q111 versus Q20 genotypes. The comparison is presented for pluripotent (ESC) and neural committed progenitors (NPC). The number of small RNAs increasing (*Increasing in Q111*—upper part of the plot), and decreasing their expression in Q111 versus Q20 (*Decreasing in Q111*—lower part of the plot) is depicted.
(TIFF)

S11 Fig Transcripts presenting linear and back-splicing alterations partially overlap with targets of dysregulated miRNAs. **A-B)** The Venn diagrams report the overlap between mutant huntingtin’s dysregulated miRNA targets (4505 transcripts targets of 9 dysregulated miRNAs comparing Q20 versus Q111 *Htt* CAG genotypes in NPC, see Methods and S5 Table) and **(A)** the list of genes presenting altered linear alternative splicing between Q20 versus Q111 *Htt* CAG genotypes in NPC (1530) or **(B)** the list of genes presenting altered back-splicing between Q20 versus Q111 *Htt* CAG genotypes in NPC (457) (see Methods and S5 Table). Intersection enrichment p-values are calculated by Fisher’s test and shown at the bottom of each diagrams.
(TIFF)

S12 Fig Validation of selected post-transcriptional regulators altered by *Htt* CAG expansion in mouse neuronal progenitors (NPC). **A)** Dot plots and bar graphs report the RT-qPCR results of the validation for *Upf1* and *Mettl3* selected post-transcriptional splicing/back-splicing regulators (see also Fig 5). RT-qPCR assay and quantification were performed on RNA from Q20 and Q111 NPCs. Bar graphs plot the relative normalized mRNA expression levels (2^-^ΔΔCt^, see methods). Actβ *Pgk1* was using as stable housekeeping gene. Error bars represent standard deviations from the mean of 3 biological replicates. *p-value < 0.01 (Student’s unpaired t-test; *n =* 3). **B)** Representative western blot (WB) analysis reports the expression of Ptbp1, Ptbp2 and Ptbp3 proteins, normalized on Hsp90 housekeeping gene in Q20 and Q111 mNPCs. Molecular weights of proteins are indicated. **C)** The bar graph reports the quantification of the Ptbps proteins as detected by WB. While Ptbp1 and Ptbp3 are significantly less expressed in Q111 compared to Q20 NPCs, Ptbp2 is significantly more expressed in the HD condition. **p-value < 0.01, ***p-value < 0.001 (Student’s unpaired t-test; *n* = 4) **D)** The graph describes the relative normalized mRNA expression levels (2^-^ΔΔCt^, see methods). *Pgk1* was used as stable housekeeping gene. Error bars represent standard deviations from the mean of biological replicates. *p-value < 0.05 (Student’s unpaired t-test, *n* = 3). The results show that only Ptbp3 is significantly overexpressed in Q111 NPCs. **E)** The bar plot reports the RPM related to *Ptbp1*, *2* and *3* as detected in RNAseq data. These results confirmed the expression of Ptbps detected through RT-qPCR.
(TIFF)

S1 Table Related to Fig 1. The spreadsheets (n = 5) report the total description of the alternative splicing (AS) events observed in **1.** the striatum (STR), **2.** the cortex (CTX) and **3.** liver. All data are calculated comparing *Htt* CAG expanded genotypes (Q80, Q92, Q111, Q140, Q175) to Q20 control (see methods). The GO terms and pathways enrichment for **4.** AS events in the STR and **5.** Skipped exon (SE) events in the STR are presented.
(XLSX)

S2 Table Related to Fig 2. The spreadsheets (n = 10) report the total description of the alternative splicing (AS) events observed in **1.** The mouse embryonic stem cells (ESC), **2.** The mouse neuronal progenitors (NPC). All data are calculated comparing *Htt* CAG expanded genotypes (Q50, Q92, Q111) to Q20 control (see methods). The total list of transcriptionally dysregulated genes (DEG) is presented for the NPC comparisons of **3.** Q20 versus Q50, **4.** Q20 versus Q92, **5.** Q20 versus Q111. The list of GO terms and pathways enrichments for **6.** the total AS events and **7.** the skipped exon (SE) events detected between NPC Q20 versus Q111 comparison is presented. The list of GO terms and pathways associated to DEG for the 3 NPC comparisons of **8.** Q20 versus Q50, **9.** Q20 versus Q92, **10.** Q20 versus Q111 is accessible. Highlighted in yellow pathways and terms related to RNA binding proteins (RBPs) and RNA modifications.
(XLSX)

S3 Table Related to Fig 3. The spreadsheets (n = 4) report the **1.** list of genes affected by skipped exons (SE) events in the striatum (STR, all genotypes and time-points pooled together), in the mouse neuronal progenitors (NPC, all genotypes) and the intersection between these two groups as presented in Fig 3A. The list of **2.** GO terms and **3.** pathways associated to the genes belonging to the intersection of Fig 3A is presented. **4.** The list of binding motifs characterizing upstream and downstream regions, adjacent to SE events in the STR and NPC is reported.
(XLSX)

S4 Table Related to Fig 4. The spreadsheets (n = 12) report **1.** the circRNA and **2.** Linear RNAs count in mouse neuronal progenitors (NPC) and **3. 4.** embryonic stem cells (ESC) from the *in vitro Htt* CAG expanded alleles. Data are presented for all genotypes (Q20, Q50, Q92, Q111) for biological duplicate experiments. **5.** Total list of dysregulated circRNAs in ESC and NPC. The comparison between the two most extreme *Htt* CAG expanded alleles (Q20 versus Q111) is reported. The list of **6.** GO terms and **7.** pathways associated to the genes originating differentially expressed circRNAs from the NPC Q20 versus Q111 comparison reported in Fig 4A is presented. **8.** List of the 12 circRNAs satisfying the 3 stringent criteria of decreasing expression and negative correlation with *Htt* CAG, significantly different expression by circTest (see Methods for further details) is presented. CircRNA identification (ID), Chromosomal location (Chr), genomic coordinates, strand, overall circularization region and circRATIO [ratio circRNA / (circRNA+linear)] is reported for each molecule. **9.** CISBP and **10.** POSTAR motif analysis (see methods) of the sequences adjacent (100bp +/-) to circularizing points for the 12 stringently defined circRNAs (Fig 4D). **11.** Mbnl1 CLIP biding sites on the 12 stringently defined circRNAs (Fig 4D). **12.** The total list of transcriptionally dysregulated (DE) small RNA is presented for the ESC and NPC comparisons for the Q20 versus Q111.
(XLSX)

S5 Table Related to Fig 5. The spreadsheets (n = 12) report the list of post-transcriptional regulators as **1.** RNA binding proteins (RBPs), **2.** Splicing factors, **3.** Factors implicated in non-sense mediated decay (NMD) and **4.** Players of the N6-methyladenosine methylation pathway (m6A). The inventory of the post-transcriptional regulators significantly altered in the various *in vitro Htt* CAG expanded neuronal progenitors (NPC) is presented for **5.** Q20 versus Q111, **6.** Q20 versus Q92 and **7.** Q20 versus Q50 comparisons. **8.** The list of mutant huntingtin interactors from the study by Podvin et al., 2022 [47] is reported. **9.** Total catalogue of miRNAs (n = 9) transcriptionally altered by the expression of mutant huntingtin in the comparison between Q20 versus Q111 NPC. **10.** Record of the predicted targets of the miRNAs dysregulated in NPC Q20 versus Q111 comparison. **11.** List of dysregulated miRNAs targets genes also transcriptionally altered in NPC Q20 versus Q111 comparison. In orange the selection of transcripts annotated as RBPs, Splicing, NMDs and M6A regulators, also previously reported as mutant huntingtin interactors [47]. **12.** The list of GO terms and pathways associated to the genes belonging to the intersection of S11A Fig is presented.
(XLSX)

S6 Table Full list of primers used in the study. (XLSX)
