# Supplementary figures and images for: CAG repeat expansion in the Huntington’s disease gene shapes linear and circular RNAs biogenesis
Source: PLoS Genet. 2023 Oct 13;19(10):e1010988. doi: 10.1371/journal.pgen.1010988 (PMC10617732; doi:10.1371/journal.pgen.1010988)

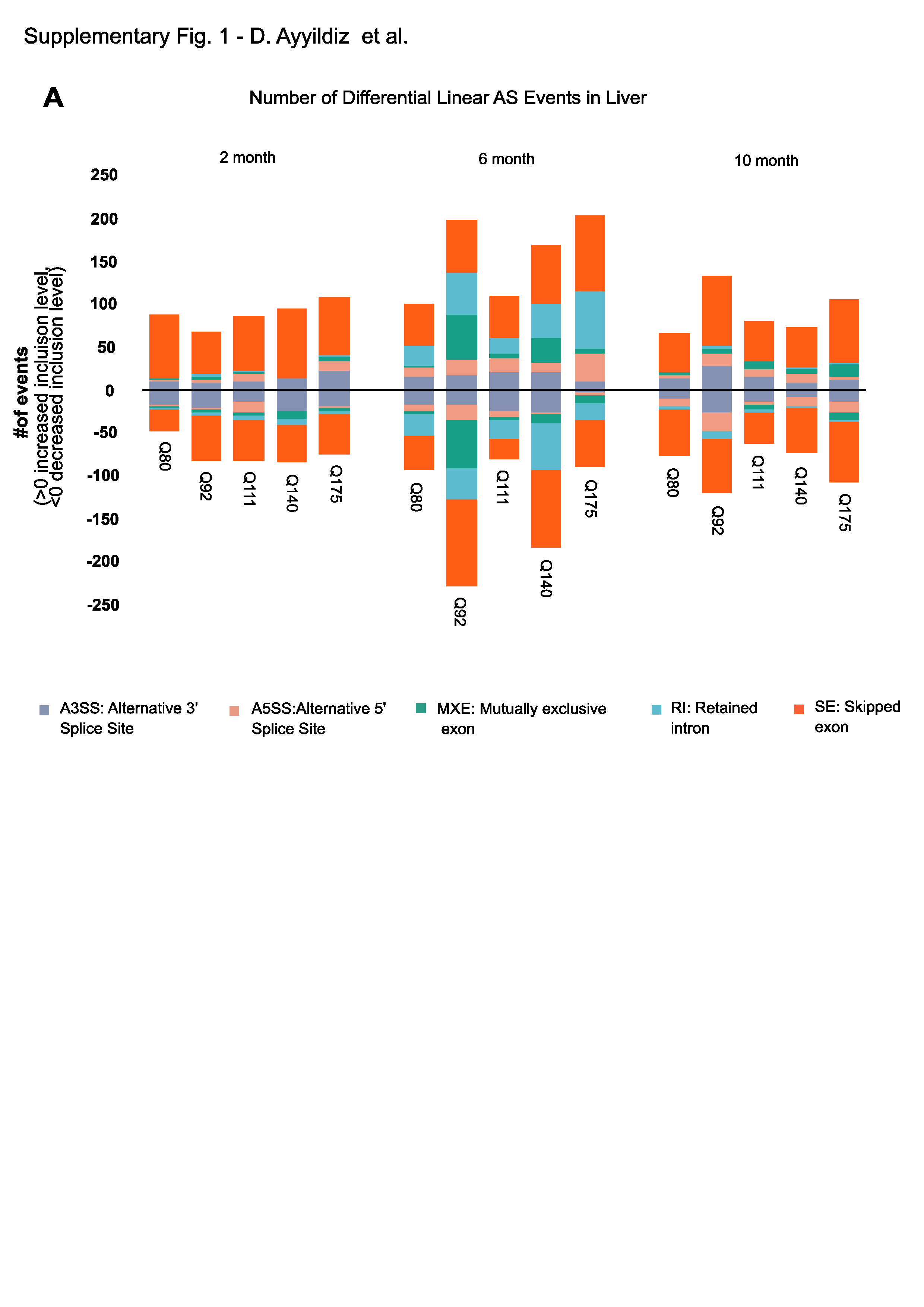

Supplement: S1 Fig — The bar graph reports the number of differential AS events in the liver from mouse KI models of HD, presenting 6 different Htt CAG repeat lengths (Q20, Q80, Q92, Q111, Q140 and Q175) and 3 time points (2, 6, and 10 months). The inclusion level is calculated in comparison to Q20 controls and the positive or negative values are plotted in the graph. The number of events is reported for each genotype and time point. Source data by Langfelder P. et al (2016) [28]. Further details can be found in the Methods section and S1 Table. Each color of the bar chart represents a different AS type. (TIFF) [file pgen.1010988.s001.tiff]

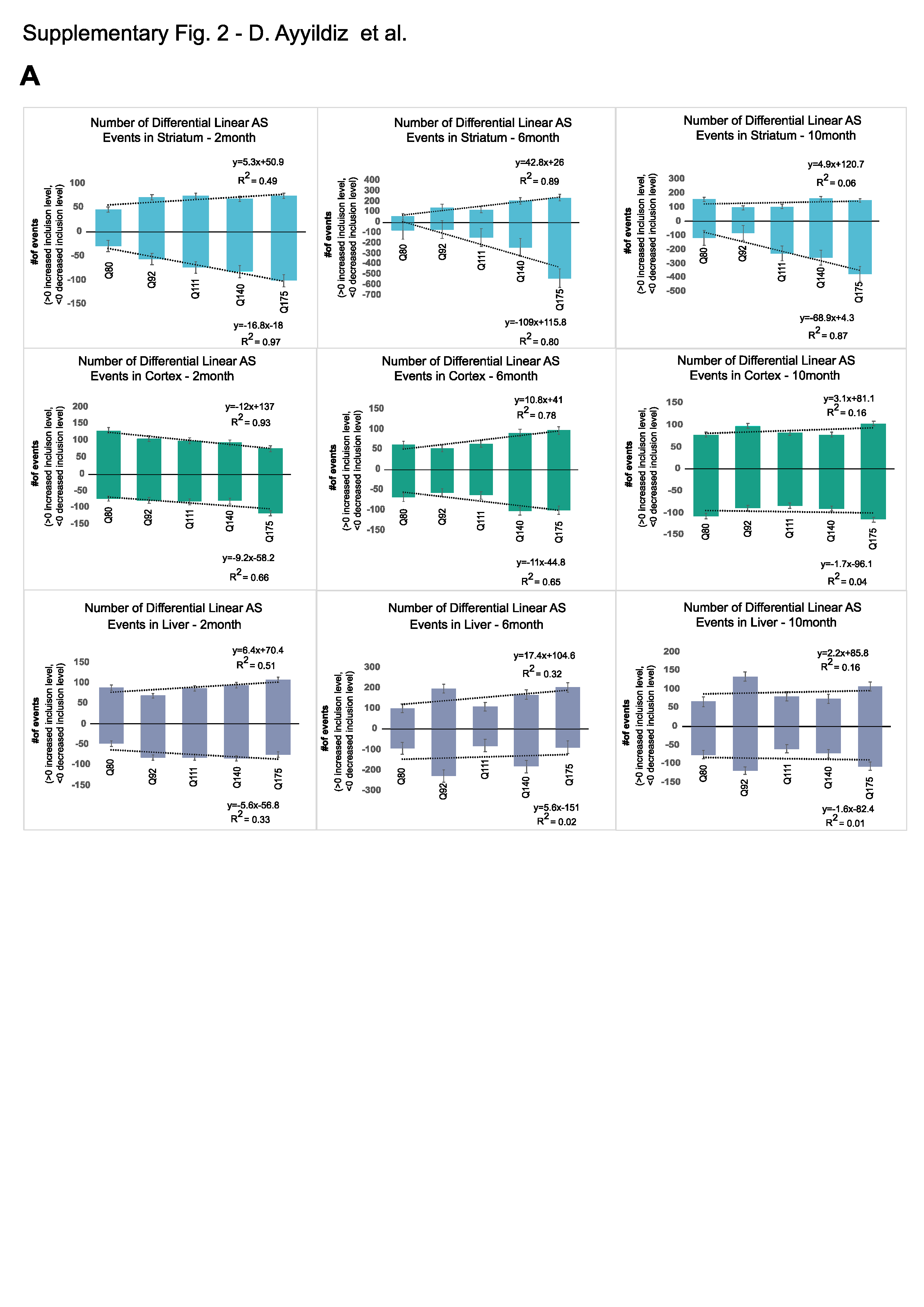

Supplement: S2 Fig — The bar graphs report the total number of differential AS events in the striatum (upper row, light blue), cortex (mid row, green) and liver (bottom, purple row) from mouse KI models of HD, presenting 6 different Htt CAG repeat lengths (Q20, Q80, Q92, Q111, Q140 and Q175) and 3 time points (2, 6, and 10 months). The inclusion level is calculated in comparison to Q20 controls and the positive or negative values are plotted in the graph. The number of events is reported for each genotype and time point (different Y-axis values are presented). Source data by Langfelder P. et al (2016) [28]. Further details can be found in the Methods section and S1 Table. The Pearson’s correlation (R2) between differential AS and Htt CAG expansion is plotted in each graph. Standard deviations and trend lines are presented. (TIFF) [file pgen.1010988.s002.tiff]

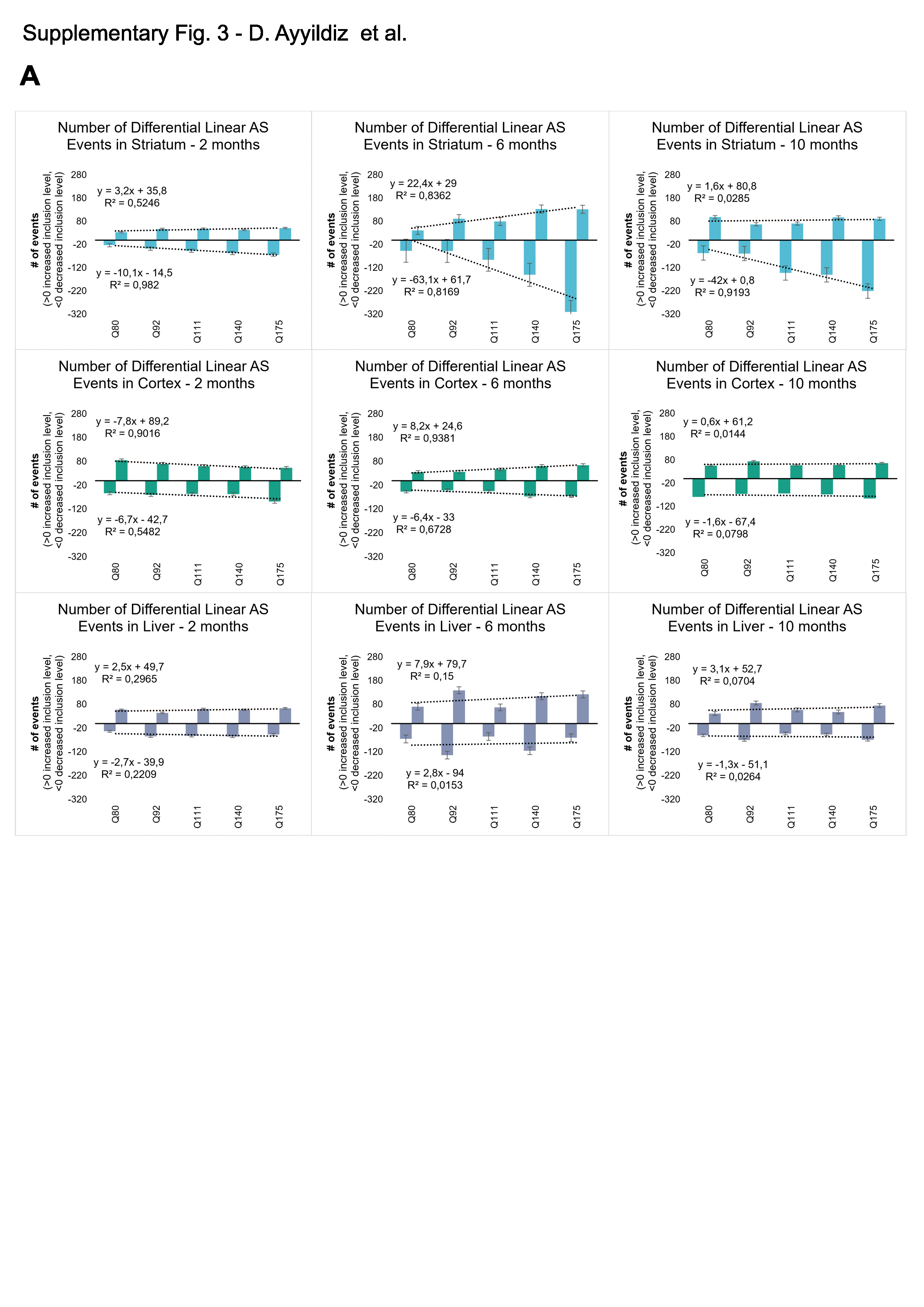

Supplement: S3 Fig — The bar graphs report the number of different alternative splicing events’ types (A3SS: Alternative 3’ splice site, A5SS: Alternative 5’ splice site, MXE: Mutually exclusive exon, RI: Retained intron and SE: Skipped exon) significantly altered in the striatum of mouse KI models of HD, presenting 6 different Htt CAG repeat lengths (Q20, Q80, Q92, Q111, Q140 and Q175) and 3 time points (2, 6, and 10 months; Blue, Orange and Green, respectively). The inclusion level is calculated in comparison to Q20 controls and the positive or negative values are plotted in the graph. The number of events is reported for each genotype and time point. Source data by Langfelder P. et al (2016) [28]. Further details can be found in the Methods section and S1 Table. The Pearson’s correlation (R2) between different AS events’ types and Htt CAG expansion is plotted in each graph. Standard deviations and trend lines are also presented. (TIFF) [file pgen.1010988.s003.tiff]

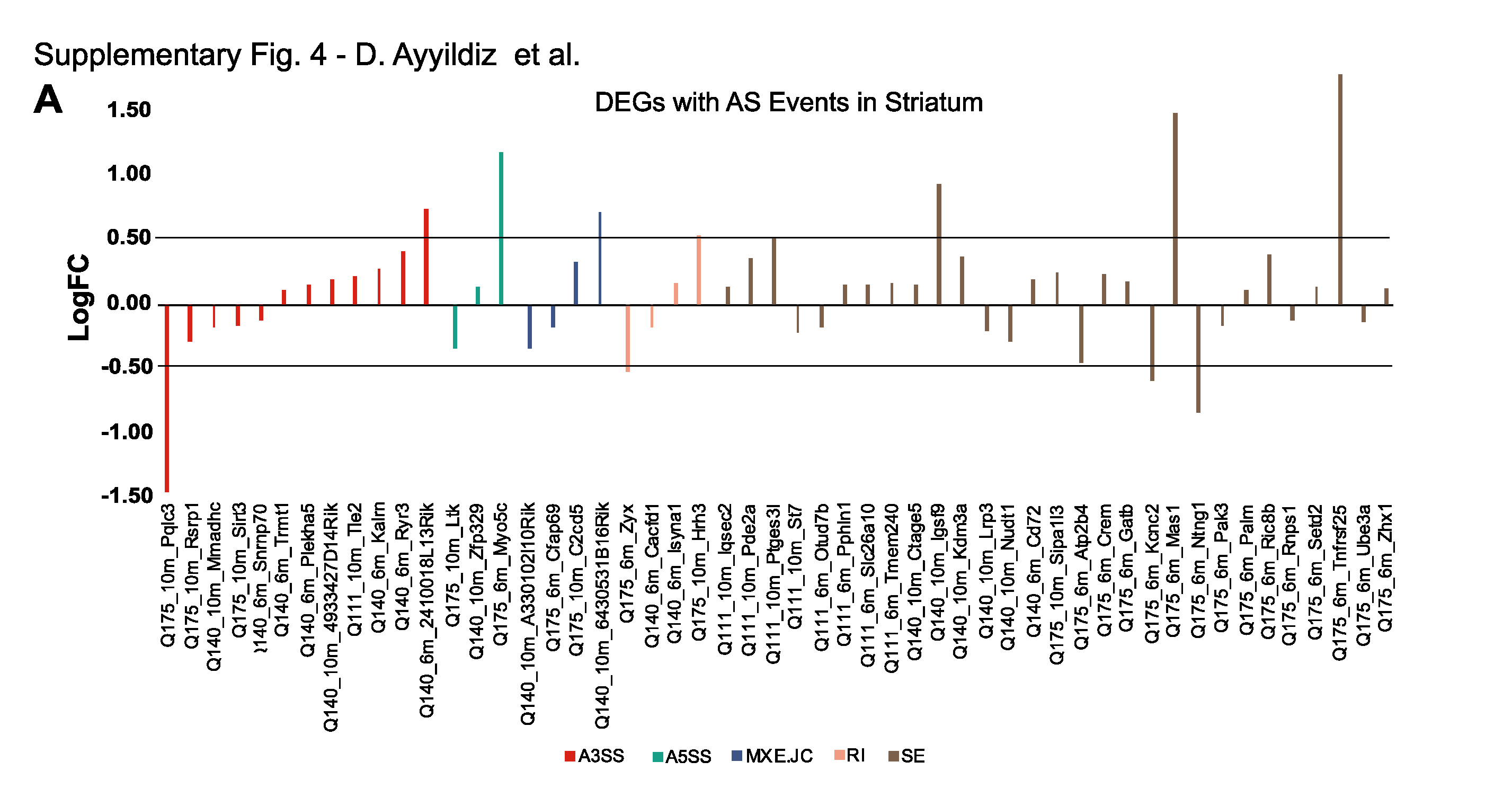

Supplement: S4 Fig — The bar graph presents the number of differentially expressed genes (DEG) which also are characterized by at least one significant AS event in the striatum of KI animal models of HD. Logarithmic fold change (LogFC) of their expression compared to Q20 control is reported in the y-axis. Transcripts are filtered based on significant p-value (p-value <0.05). Transcripts names and condition were the differential expression was observed are indicated in the x-axis. Transcripts are divided accordingly to the specific AS event type (A3SS: Alternative 3’ splice site, A5SS: Alternative 5’ splice site, MXE: Mutually exclusive exon, RI: Retained intron and SE: Skipped exon). Color code defines events types and DEG bars. (TIFF) [file pgen.1010988.s004.tiff]

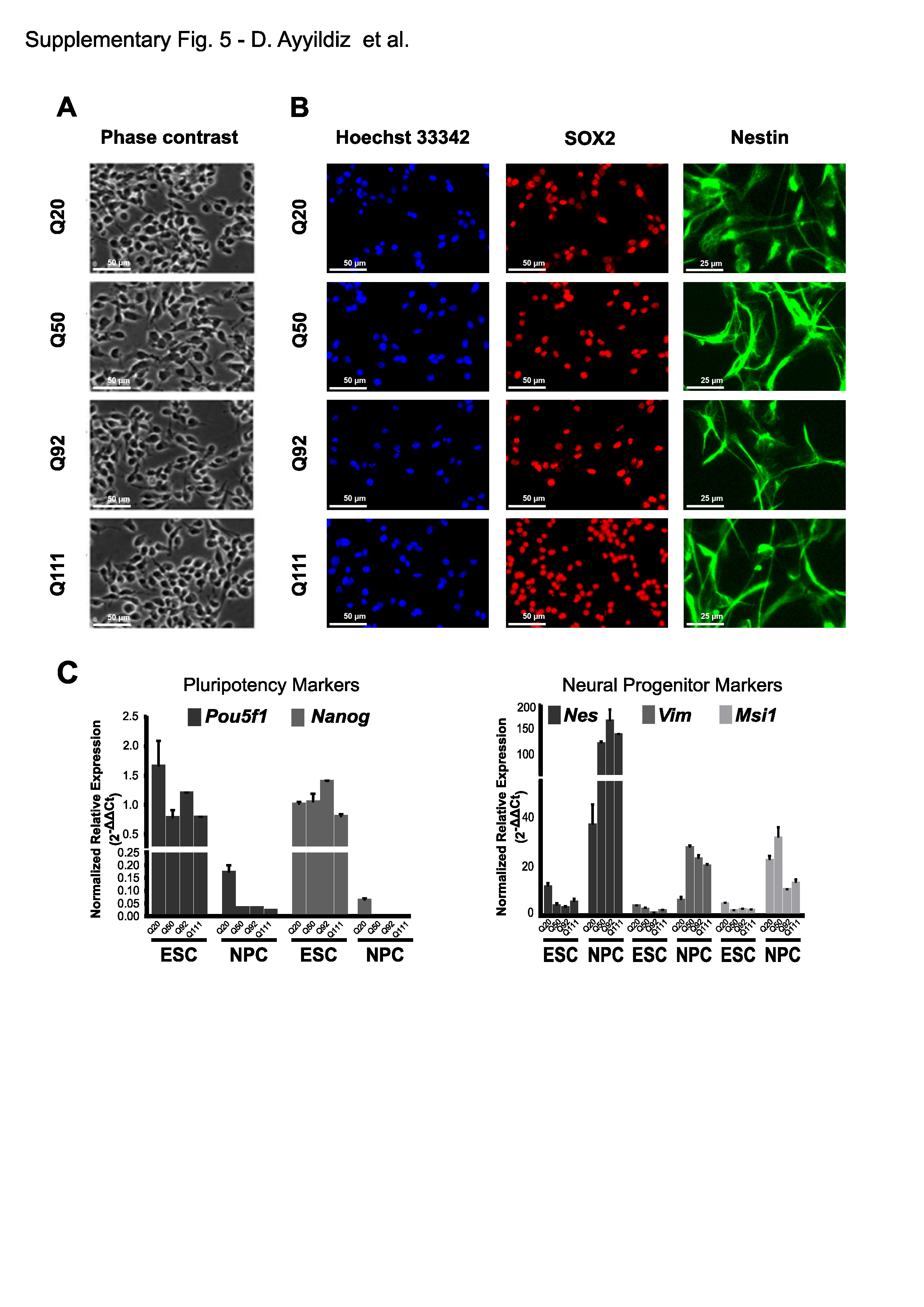

Supplement: S5 Fig — A) Phase contrast micrographs of heterozygous Htt CAG knock-in HttQ20, HttQ50, HttQ92 and HttQ111 (Q20, Q50, Q92, Q111) NPC lines derived from neural differentiation of ESC display the appropriate morphology with neurite extensions. B) Fluorescent images of cells, with Hoechst 33342 stained nuclei, show proper expression of Sox2 and Nestin neuroectodermal markers in the NPC for each genotype. Scale bars = 50 μm. Nestin is enlarged to better appreciate neuronal processes. Scale bars = 25 μm. C) Bar graphs plot the relative normalized mRNA expression levels of pluripotency marker genes Pou5f1 and Nanog and neuroectodermal marker genes Nes, Vim and Msi1 as determined by RT-qPCR amplification assays. Error bars represent standard deviations from the mean of two biological and two technical replicates. (TIFF) [file pgen.1010988.s005.tiff]

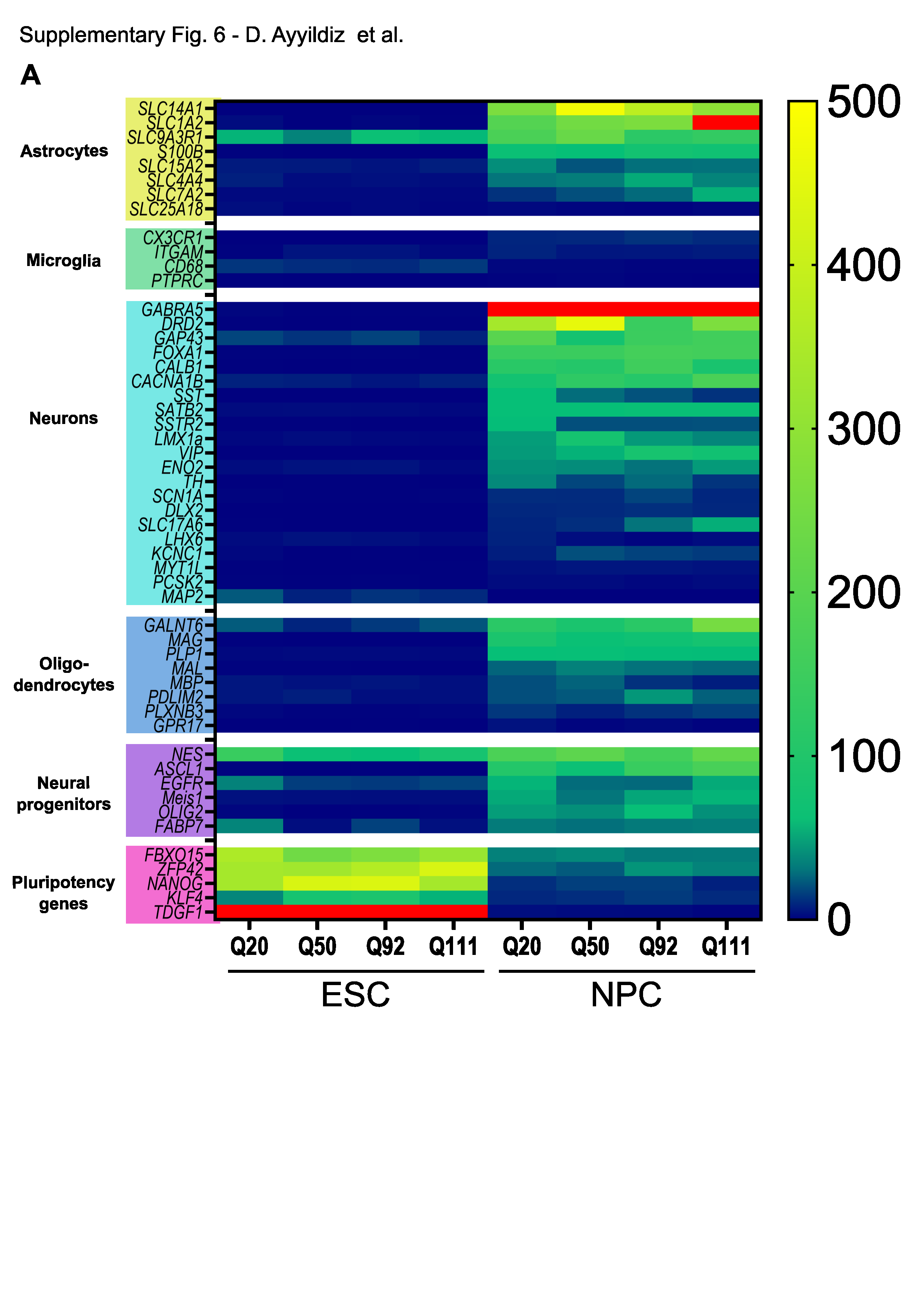

Supplement: S6 Fig — The heatmap describes the normalized counts per millions (cpm) of cell types specific markers as described in [38]. Astrocytes, microglia, neurons, oligodendrocytes, neural progenitors and pluripotent cells were analysed for heterozygous Htt CAG knock-in HttQ20, HttQ50, HttQ92 and HttQ111 (Q20, Q50, Q92, Q111) in their transition from ESC to NPC. This deep transcriptional characterization confirms a similar pattern of expression between different genotypes and a still not fully committed neuronal progenitor state. (TIFF) [file pgen.1010988.s006.tiff]

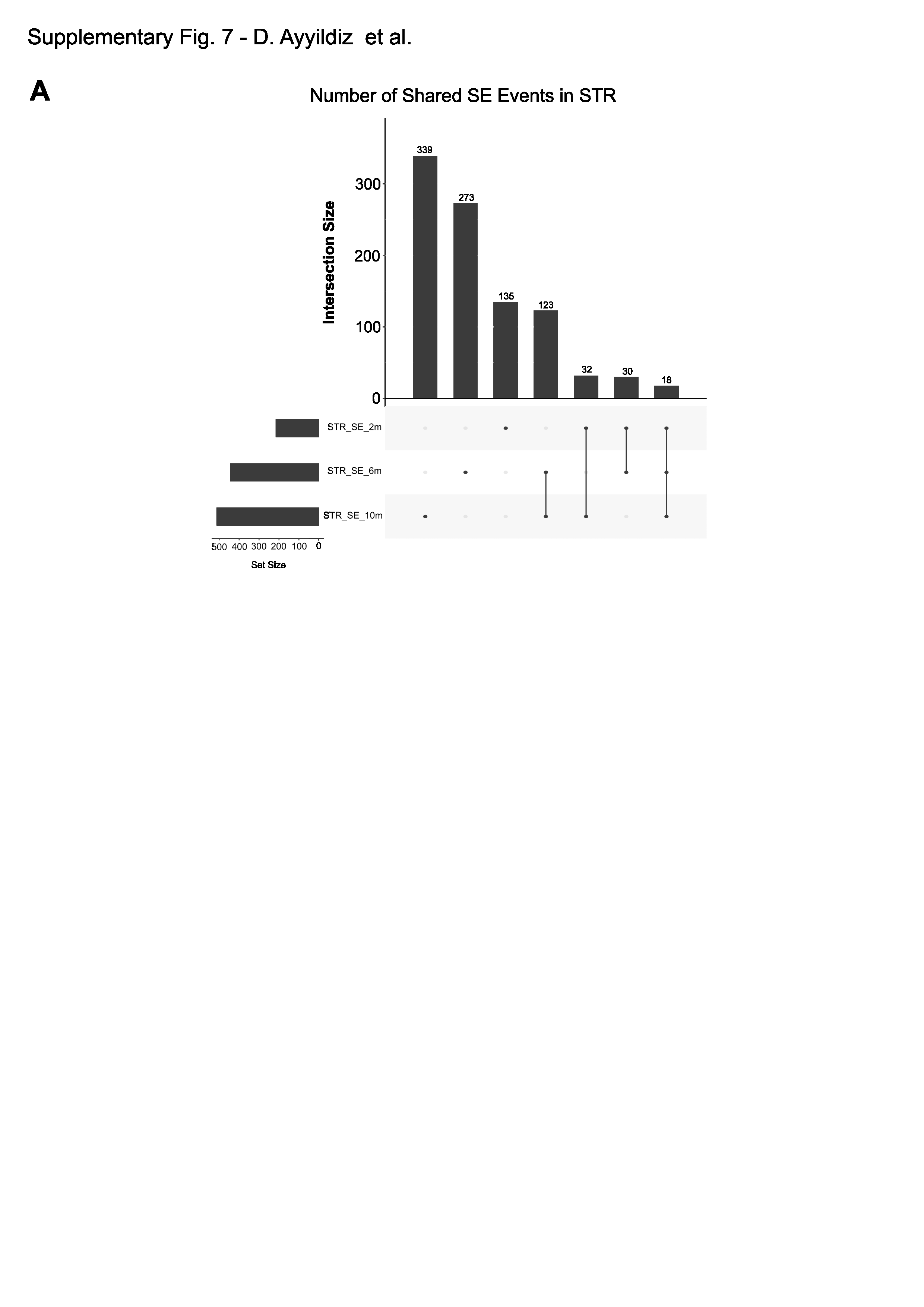

Supplement: S7 Fig — The upset plot displays the number of SE events shared among different time points (2, 6 and 10 months) within the mouse striatum. For each time points, all the KI mice genotypes with expanded CAG tract were pooled together. The number of events within each intersection is presented in the vertical bars. Intersection groups (lines) or single time points (dots) are shown in the lower panel. Sample set size is indicated at the bottom left of the panel. (TIFF) [file pgen.1010988.s007.tiff]

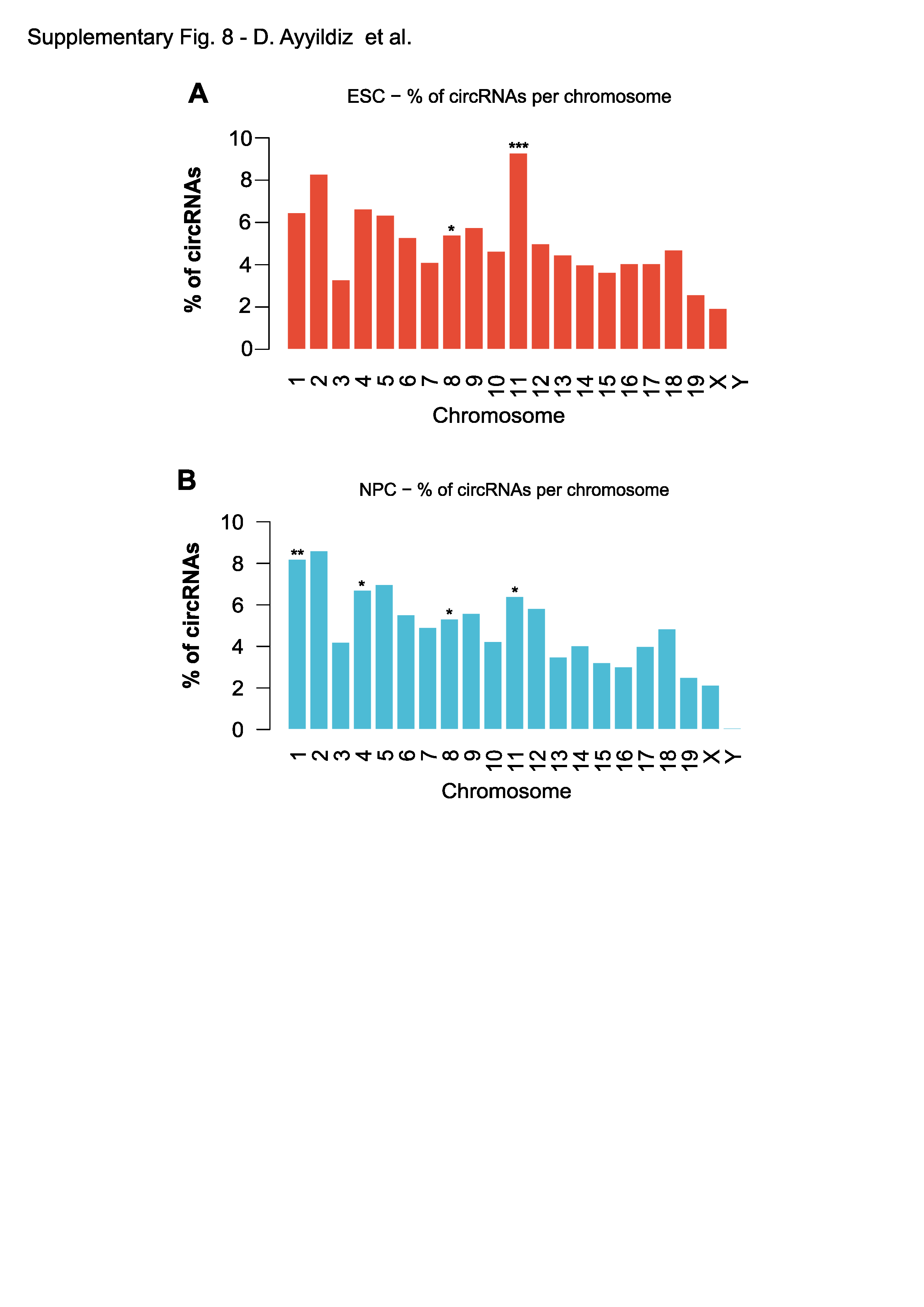

Supplement: S8 Fig — A-B) The bar charts display the percentage of detected circRNAs originating from each chromosome in ESC (A) and NPC (B). Enrichment of circRNAs, using the number of protein-coding genes contained in each chromosome as a background, was computed by a Fisher test and significant p-values are displayed over the graph bars (* < 0.05, ** < 0.01, *** < 0.001). (TIFF) [file pgen.1010988.s008.tiff]

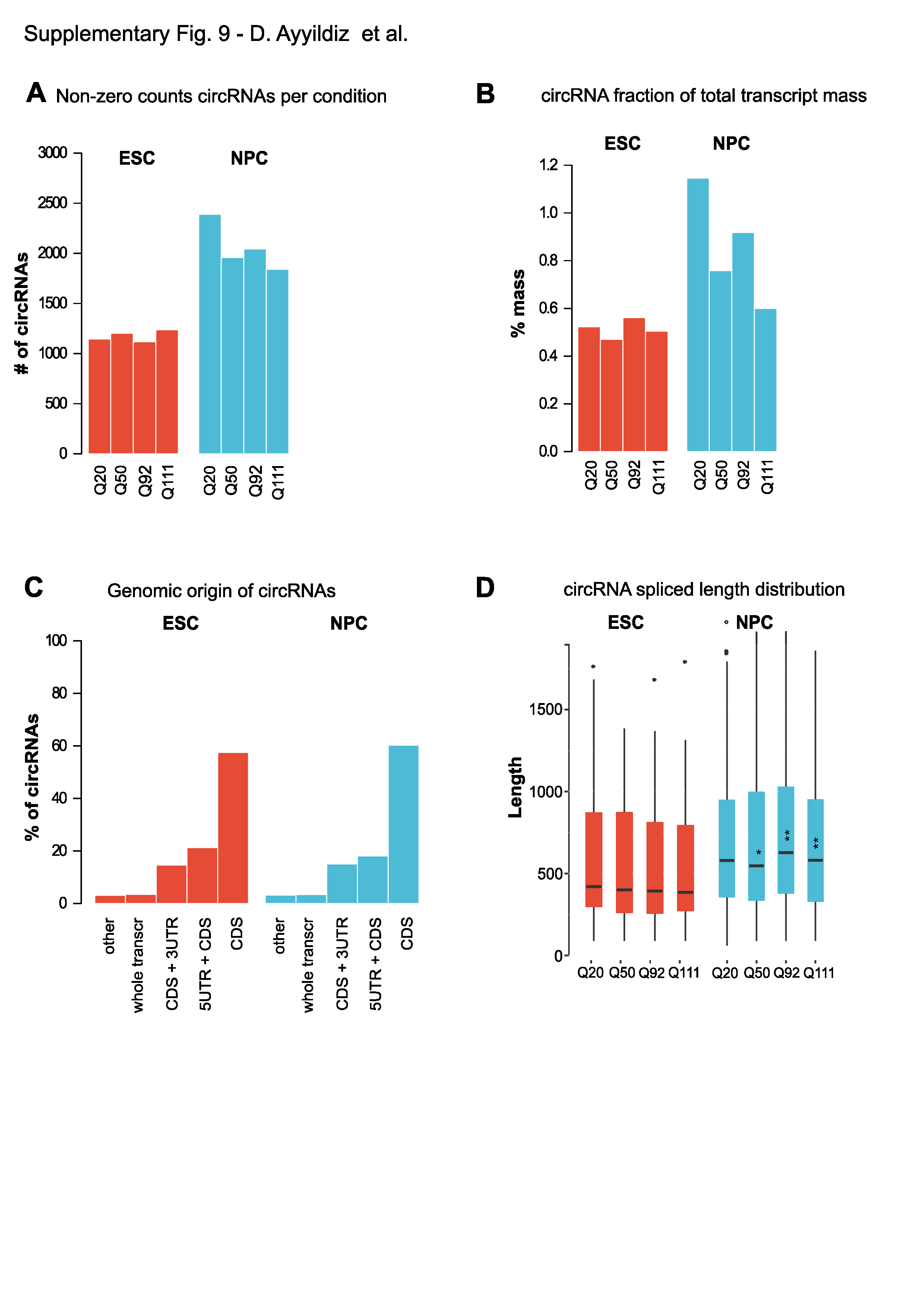

Supplement: S9 Fig — A) The bar chart shows the number of detected circRNAs (>1 count/sample, see also Methods), comparing pluripotent (ESC) and neural committed progenitors (NPC). Series of 4 Htt CAG expansion alleles (Q20, Q50, Q92 and Q111) are presented. Average circRNA count from two biological replicate experiments is plotted. B) Bars chart presents the circRNA fraction of total transcript mass in ESC and NPC different genotypes. C) The bars chart reports the percentage of circRNAs derived from specific transcripts areas: transcript coding sequence (CDS), 3’ or 5’ untranslated regions (3UTR/5UTR), whole transcript or other. The data compare ESC and NPC conditions. All Htt genotypes were combined. D) The box plot displays the spliced length distribution (in base pairs, bp) [average ± standard deviation (SD)] for circRNAs in the various conditions of ESC and NPC cells. Htt genotypes as in a). Wilcoxon test p-values of the difference between corresponding conditions of ESC and NPC are shown as stars (* < 0.05, ** < 0.01, *** < 0.001). (TIFF) [file pgen.1010988.s009.tiff]

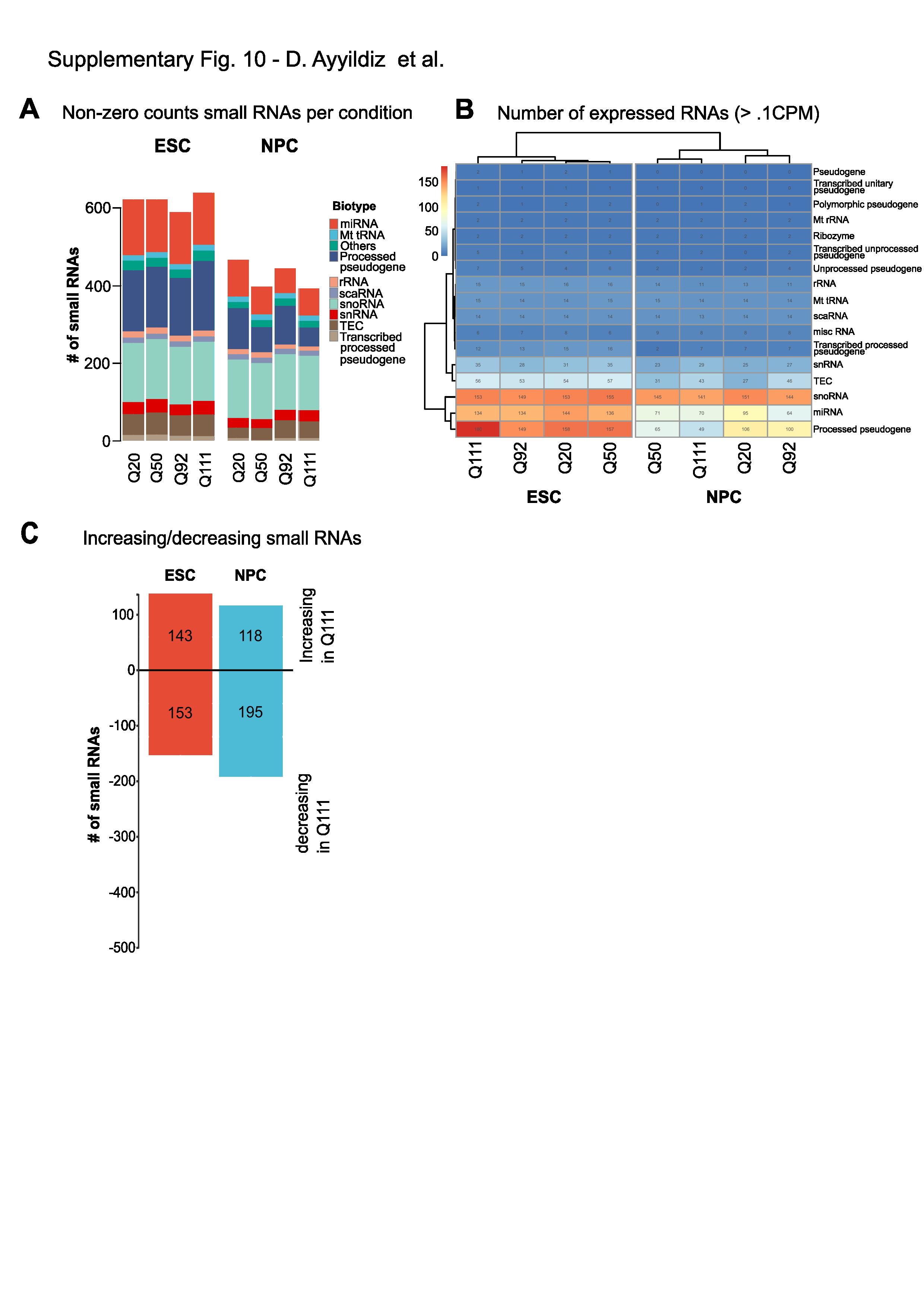

Supplement: S10 Fig — A) The coloured bar graphs report the number of small RNAs (>1 count/sample, see also Methods) comparing ESC and NPC conditions. Series of 4 Htt CAG expansion alleles (Q20, Q50, Q92 and Q111) are presented. Different classes of small RNAs are examined. Abbreviations as follows: microRNAs (miRNAs), Mitochondrial transfer RNAs (Mt-tRNAs), processed pseudogenes, ribosomal RNAs (rRNAs), small nucleolar RNAs (snoRNAs), small nuclear RNAs (snRNAs) small Cajal body-specific RNAs (scaRNAs), To be Experimentally Confirmed (TEC), transcribed processed pseudogenes. B) The heatmap presents the number of expressed (> 1 count per million, CPM) small RNAs among heterozygous Htt CAG knock-in HttQ20, HttQ50, HttQ92 and HttQ111 (Q20, Q50, Q92, Q111), comparing ESC and NPC lines. Different classes of small RNAs are examined. Abbreviations as in GENCODE transcript biotypes [86]. Color code bar (upper right) reports the number of expressed small RNAs in each condition. C) The bar chart shows the number of small RNAs differentially expressed between Htt Q111 versus Q20 genotypes. The comparison is presented for pluripotent (ESC) and neural committed progenitors (NPC). The number of small RNAs increasing (Increasing in Q111—upper part of the plot), and decreasing their expression in Q111 versus Q20 (Decreasing in Q111—lower part of the plot) is depicted. (TIFF) [file pgen.1010988.s010.tiff]

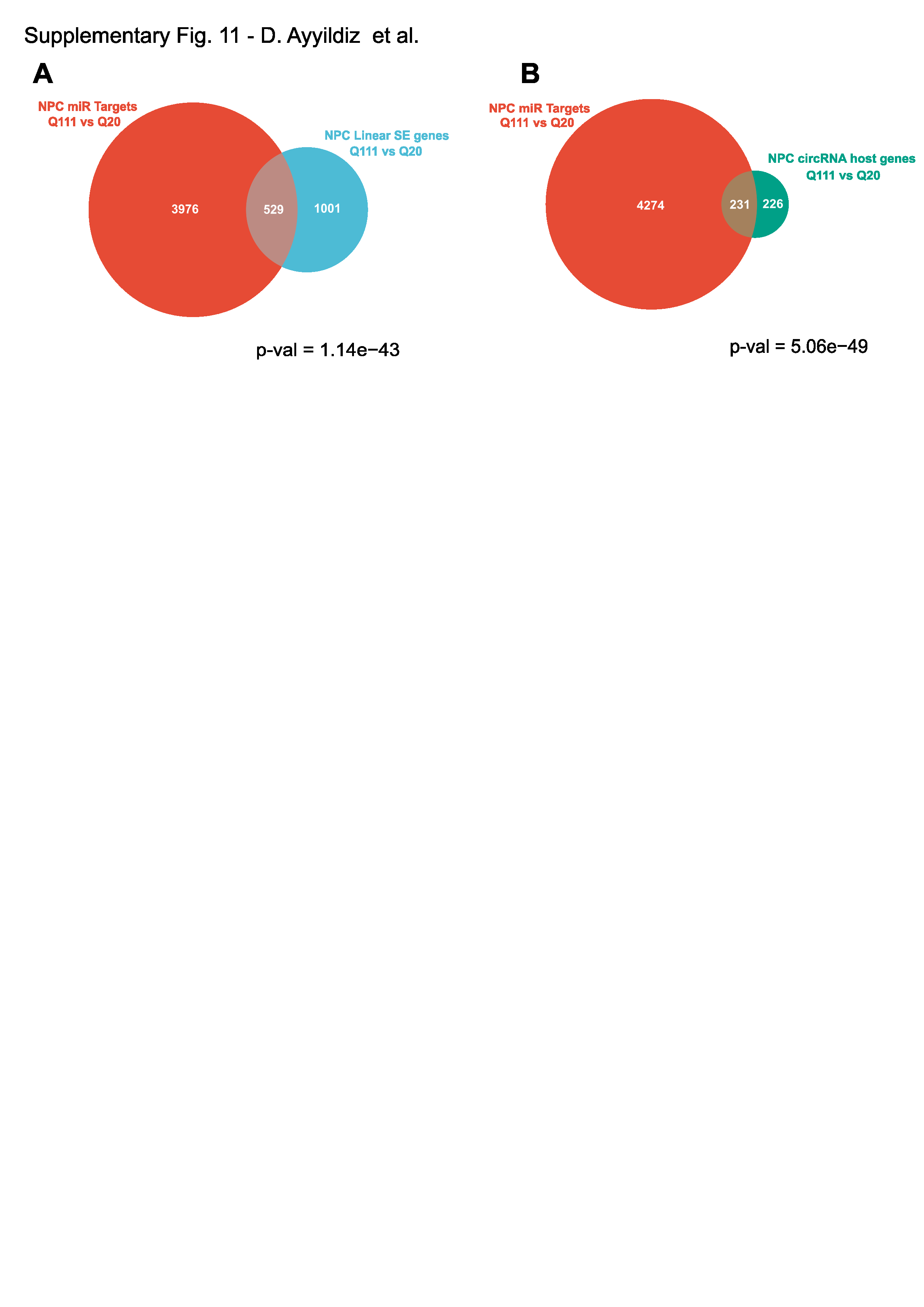

Supplement: S11 Fig — A-B) The Venn diagrams report the overlap between mutant huntingtin’s dysregulated miRNA targets (4505 transcripts targets of 9 dysregulated miRNAs comparing Q20 versus Q111 Htt CAG genotypes in NPC, see Methods and S5 Table) and (A) the list of genes presenting altered linear alternative splicing between Q20 versus Q111 Htt CAG genotypes in NPC (1530) or (B) the list of genes presenting altered back-splicing between Q20 versus Q111 Htt CAG genotypes in NPC (457) (see Methods and S5 Table). Intersection enrichment p-values are calculated by Fisher’s test and shown at the bottom of each diagrams. (TIFF) [file pgen.1010988.s011.tiff]

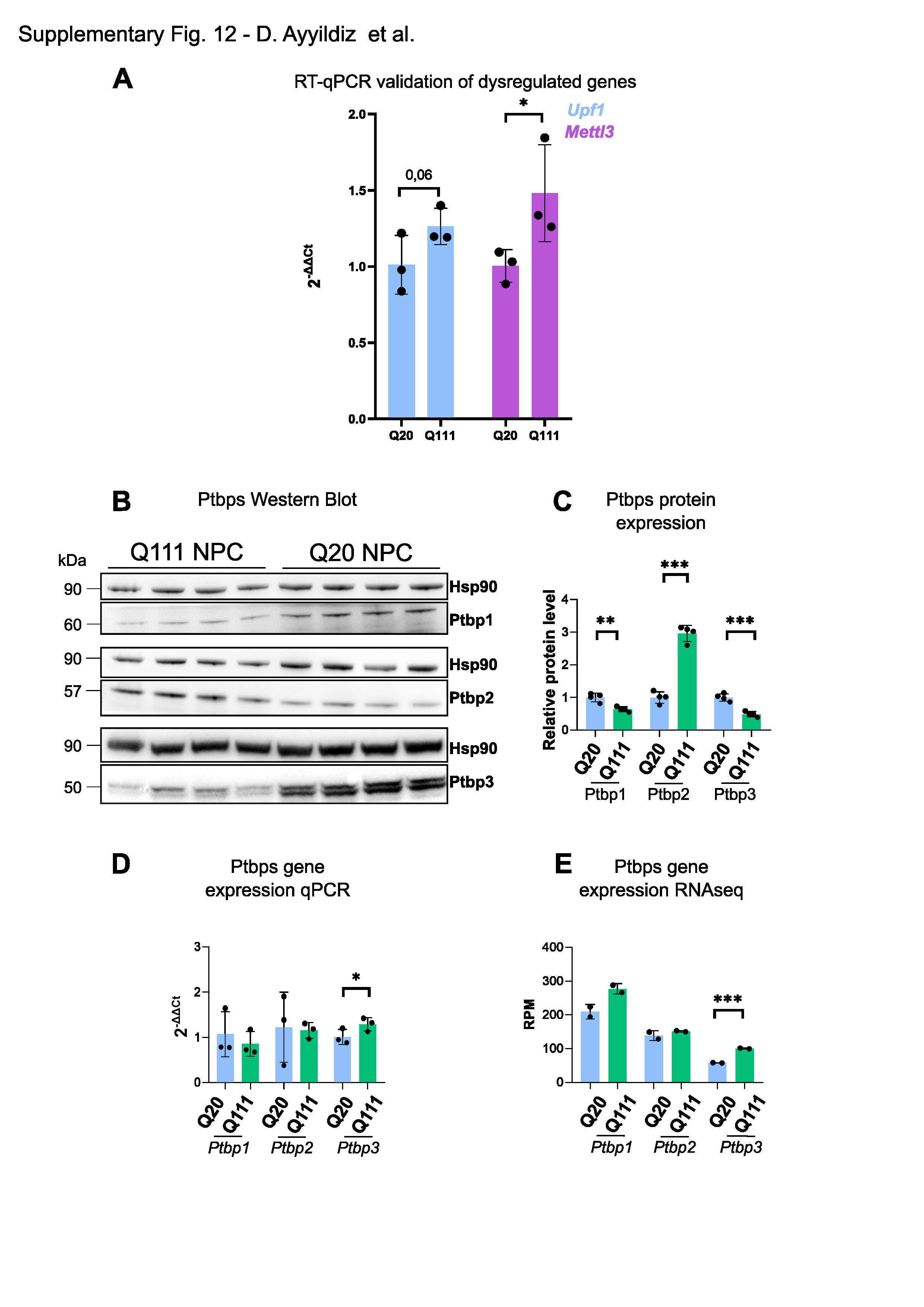

Supplement: S12 Fig — A) Dot plots and bar graphs report the RT-qPCR results of the validation for Upf1 and Mettl3 selected post-transcriptional splicing/back-splicing regulators (see also Fig 5). RT-qPCR assay and quantification were performed on RNA from Q20 and Q111 NPCs. Bar graphs plot the relative normalized mRNA expression levels (2-^ΔΔCt, see methods). Actβ Pgk1 was using as stable housekeeping gene. Error bars represent standard deviations from the mean of 3 biological replicates. *p-value < 0.01 (Student’s unpaired t-test; n = 3). B) Representative western blot (WB) analysis reports the expression of Ptbp1, Ptbp2 and Ptbp3 proteins, normalized on Hsp90 housekeeping gene in Q20 and Q111 mNPCs. Molecular weights of proteins are indicated. C) The bar graph reports the quantification of the Ptbps proteins as detected by WB. While Ptbp1 and Ptbp3 are significantly less expressed in Q111 compared to Q20 NPCs, Ptbp2 is significantly more expressed in the HD condition. **p-value < 0.01, ***p-value < 0.001 (Student’s unpaired t-test; n = 4) D) The graph describes the relative normalized mRNA expression levels (2-^ΔΔCt, see methods). Pgk1 was used as stable housekeeping gene. Error bars represent standard deviations from the mean of biological replicates. *p-value < 0.05 (Student’s unpaired t-test, n = 3). The results show that only Ptbp3 is significantly overexpressed in Q111 NPCs. E) The bar plot reports the RPM related to Ptbp1, 2 and 3 as detected in RNAseq data. These results confirmed the expression of Ptbps detected through RT-qPCR. (TIFF) [file pgen.1010988.s012.tiff]
